# Inhibition of tyrosine kinase BMX increases cell death in response to existing chemotherapeutic agents overcoming apoptotic resistance in cancer

**DOI:** 10.1038/s41419-025-08131-9

**Published:** 2025-11-10

**Authors:** Dalal Morad, Sam Skinner, Karen Bowman, Chitra Seewooruthun, Joanna L. Fox

**Affiliations:** 1 Leicester Institute of Structural and Chemical Biology, University of Leicester, Leicester, UK; 2 Leicester Drug Discovery and Diagnostics, University of Leicester, Leicester, UK; 3 Present Address: Department of Microbial Biotechnology, University of Nottingham, Nottingham, UK; 4 Present Address: School of Applied Science, University of Huddersfield, Huddersfield, UK

**Keywords:** Breast cancer, Target validation

## Abstract

BMX tyrosine kinase has emerged as a novel drug target, the inhibition of which simultaneously decreases proliferative signaling and increases cell death. BMX expression is upregulated in numerous cancers and causes acquired resistance to chemotherapeutic drugs. In addition to being responsive to multiple signaling pathways involved in regulating proliferation, migration, and cell survival, BMX is also a direct and potent negative regulator of BAK that mediates apoptotic resistance. Knockdown of BMX in cancer cells dramatically potentiates BAK activation following DNA damage, rendering cells hypersensitive to killing by otherwise sub-lethal doses of drug irrespective of the mode of drug action. This study aimed to determine whether existing BMX inhibitors could increase cell killing when used in combination with chemotherapeutic agents. We now show this sensitization can be phenocopied using small molecules BMX inhibitors, confirming inhibition of regulators upstream of the apoptotic machinery is a novel strategy to modulate cell death and improve the efficacy of existing chemotherapeutic agents. Analysis of the binding of existing BMX inhibitors BMX-IN-1 and CHMFL-BMX-078 to the BMX kinase domain reveals these molecules’ potential limitations, including the high cross-reactivity with other members of the TEC kinase family. This study serves as proof-of-principle for this therapeutic strategy but also highlights the urgent need for more specific molecules to inhibit BMX kinase activity.

**Figure:**
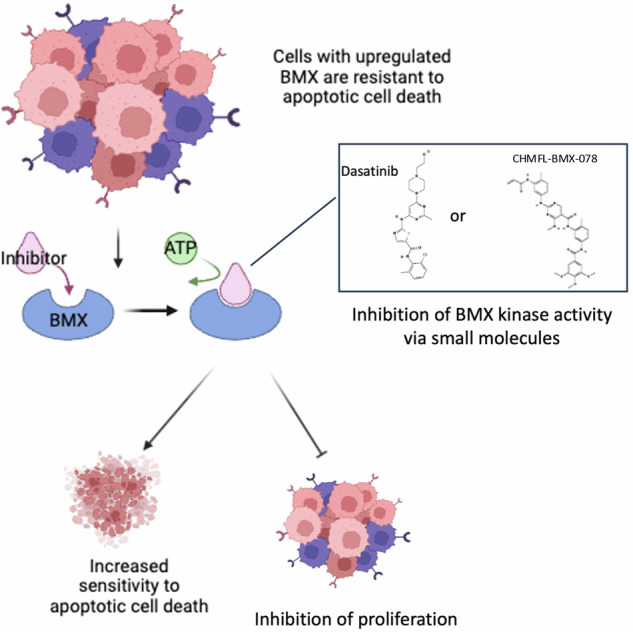
Created with BioRender.com.

Created with BioRender.com.

## Introduction

Resistance to cell death is a hallmark of cancer [1] and a major challenge in developing effective treatments. The core apoptosis machinery is often inactivated in cancer, allowing cells to evade apoptosis via various mechanisms, including modifying upstream regulatory signalling that regulates apoptosis. One such change is upregulation of BMX tyrosine kinase, which, although not a core component of the apoptosis machinery, has been found to play a critical role in the cell fate decision to initiate apoptosis in response to chemotherapy [2].

Upregulation of BMX is implicated in the development of numerous cancers, including Bladder, Renal Cell Carcinoma, Triple Negative Breast Cancer (TNBC), Prostate, and Castration-Resistant Prostate Cancer (CRPC) [2–4]. BMX has multiple cellular functions, including increasing proliferation, inhibition of apoptosis, induction of cytokine secretion, and induction of inflammatory signalling [5], all of which can contribute to tumorigenesis. BMX is a member of the TEC family of non-receptor tyrosine kinases, which includes TEC, BTK, ITK, and TXK [6]. BTK and ITK are also upregulated in cancer and are well-validated drug targets [7, 8]. There are four FDA-approved BTK inhibitors available for blood malignancies [9] and several ITK inhibitors currently in clinical trials [10]. However, to date, no BMX inhibitors have progressed to clinical trials.

Like all the TEC kinases, BMX contains five structural domains; a pleckstrin homology (PH) domain required for membrane localisation, a zinc-binding BTK homology (BH) motif, two Src homology domains (SH3 and SH2), which regulate the kinase activity of the protein, and a kinase domain [6]. Structurally, the ATP-binding kinase domain of BMX is akin to all the other TEC kinases, but has the closed sequence homology with BTK. The N-terminal lobe (residues 408-497) is comprised of β-strands with a single αC helix (454–466), a G-loop (417–436), and a hinge region (490–497). The C-terminus (498–671) is comprised of α-helices, a DFG motif (554–556), and an activation loop (557–576) [3]. Due to this high level of similarity between BMX and BTK, designing a selective BMX inhibitor is challenging, with many of the existing BTK inhibitors targeting BMX with a similar potency. Additionally, although structures of the PH, SH2, and kinase domains of BMX have been solved by NMR and X-ray crystallography, respectively, the full-length structure of BMX has not been determined; therefore, inter-domain interactions that contribute to its regulation remain uncharacterised.

BMX has many known substrates whose activity is modulated in cancer due to BMX overexpression. In breast cancer BMX overexpression promotes proliferation by upregulating the activity of downstream substrates Protease Activated Receptor 1 (PAR1), p21 Activated kinase 1 (PAK1), and Signal Transducer and Activator of Transcription 1 (STAT1; [11]). BMX upregulation can also drive fibroblast transformation via Signal Transducer and Activator of Transcription 3 (STAT3) in ductal breast cell transformation by increased PAK1 signalling [12]. Furthermore, phosphorylation of its substrates at pYY sites, increases their activity and leads to enhanced RAS, SRC, and PI3K signalling, which in turn further upregulates BMX [13]. Alternatively, BMX can block apoptosis via several mechanisms; in LNCaP prostate carcinoma cells, BMX inhibits the p53 DNA damage response [14]. For the purpose of this study, we have focused on BMX’s role in downregulating apoptotic cell death via its ability to inhibit activation of BCL-2 family effector protein BAK, whose role in apoptosis is to permeabilise the outer mitochondrial membrane. Specifically, phosphorylation of tyrosine residue 108 (Y108) by BMX renders BAK inactive [2, 15], maintaining BAK in its inactive conformation and cannot be ‘auto-activated’, be activated by BH3-only proteins, or form oligomeric structures. This increases the cell’s ability to withstand apoptotic signalling and facilitates evasion of cell death. In normal cells, BMX and BAK exist in a stable complex that dissociates once apoptotic stimuli are received [2]. However, cancer cell survival signalling upregulates BMX activity and phosphorylation of BAK at Y108, meaning the apoptotic threshold is held abnormally high. This contributes to the ability of the cancer cell to evade cell death, hence why high BMX overexpression is known to confer resistance to chemotherapeutic agents [16].

Although BMX has emerged as an attractive cancer target, genetic studies revealed that removing BMX kinase activity alone is insufficient to kill the cancer cell, and BMX knockout in mice is viable [17]. However, knockdown of BMX greatly sensitises cancer cells to standard chemotherapeutic agents [2]. The current study aims to determine whether small-molecule inhibitors of BMX in combination with existing chemotherapeutic agents can phenocopy the previously observed sensitisation. This is supported by structural analysis of the binding of existing inhibitors to BMX in combination with cell-based analysis of efficacy to identify the most impactful interactions with the catalytic pocket to drive improvement and development of future BMX inhibitors.

## Materials and Methods

### Cell culture

HT1080, MDA-MB-231, MDA-MB-468, and MCF7 cells were obtained from and characterized by ATCC. All cells were mycoplasma-free when tested with EZ-PCR™ Mycoplasma Detection Kit (Satorius) and used for less than 3 months of continuous passage.

### Chemicals and drug treatments

All chemicals, except where specified, were purchased from Calbiochem and used at the final concentrations indicated. Treatments with Camptothecin (CPT; 6 μM), Etoposide (ET; 5 μM), BMX-IN-1 (20 µM), CHMFL-BMX-078 (20 μM), and ABT-737 (20 μM) were carried out at various time points depending on the assay.

### Cell growth inhibition studies

MTT cell growth assays were used to determine viable cells according to the manufacturer’s instructions (475989; Sigma Aldrich). The IC_50_ was calculated as the drug concentration that inhibited cell growth by 50% compared with vehicle controls (<0.1% DMSO).

### Flow cytometric analysis

Flow cytometric analysis of BAK conformation was determined using Ab-1 (AM03; Calbiochem) as previously described [15]. Detection of Annexin V was used as a marker for apoptotic cells as described [1].

### Western blotting

Cells were prepared and analysed as previously described [15]. Antibodies used were as follows: anti-Btk (#3547; Cell Signaling Technology), anti-BMX (#610792; BD Bioscience), anti-pERK1/2 (#9101; Cell Signaling Technology), total anti-ERK1/2 (#9102; Cell Signaling Technology) anti-pAKT (#9271; Cell Signaling Technology), total anti-AKT (#9272; Cell Signaling Technology), anti-Vinculin (#VMA00895; BioRad). Secondary antibodies were goat anti-mouse or goat anti-rabbit IRDye fluorescently tagged (used at a dilution of 1:10,000; LiCor UK). Reactive proteins were visualised using Odessey (LiCor).

### Recombinant kinase assay

The effect of inhibitors on BMX kinase activity was determined using the ADP-Glo BMX kinase assay according to the manufacturer’s instructions (Promega #V6930) with the BMX Kinase Enzyme System (Promega #V4512).

### Protein expression

Wild-type BMX kinase domain (residues 417 to 675) was cloned into pCDNA3 with an N-terminal TEV cleavable 6xHIS tag and expressed in BL21(DE3) cells. The resultant protein was purified using a HisTrap™ excel column followed by a HiLoad® 16/600 Superdex® 75 pg column. The His-tag was cleaved using TEV. Concentrated protein was stored at −80 °C.

### Far-UV Circular Dichroism (CD) spectroscopy

Protein secondary structure was determined by CD spectroscopy with an Applied Photophysics Chirascan VX blanked with gel filtration buffer, using LAAPD detectors at 20 ^o^C for a pathlength of 10 mm in the far-UV wavelength range of 195–260 nm. Protein concentration was 0.1 mg/mL.

### Differential Scanning Fluorimetry (DSF)

DSF was performed on a MyIQ^TM^ Single Color Real-Time PCR Detection System in MicroAMP^TM^ Fast Optical 96-Well Reaction Plates. The assay mixture contained 10 μM of BMX kinase +/− 100 µM BMX inhibitor BMX-IN-1 and 5-fold SYPRO^®^ Orange Protein Gel Stain dye (491 nm excitation peak and 586 nm emission peak) in buffer (10 mM Hepes pH 8.0, 150 mM NaCl). Temperature was raised to 85 ^o^C from 25 ^o^C at increments of 0.06 ^o^C/s. Two runs were performed for BMX-IN-1; one immediately at room temperature following preparation and one after 2 h incubation at 4 ^o^C. The latter replicated the conditions used in *Seixas* et al. [18] for BMX-IN-1 and JS24.

### Compound docking

BMX-IN-1 and CHMFL-BMX-078 were docked into BMX DFG OUT (PDB: 3SXR) and BMX DFG IN (PDB: 8X2A). Docking was performed using AutoDock 4.2.6 [19]. The BMX structures included all protein atoms in chain A of the N-lobe. Non-polar hydrogens were added, and Gasteiger charges were calculated using AutoDock. Both ligands were obtained from PubChem, and their atomic Gasteiger charges were computed using AutoDockTools.

For docking into each BMX conformation, a grid map was constructed with dimensions of 126 × 100 x 100 points and a spacing of 0.2 Å. 200 runs were performed using the Lamarckian genetic algorithm with default parameters. The docked conformations were sorted by binding energy, and the model with the lowest binding energy was selected as the final docking output.

## Results

**Fig. 1:**
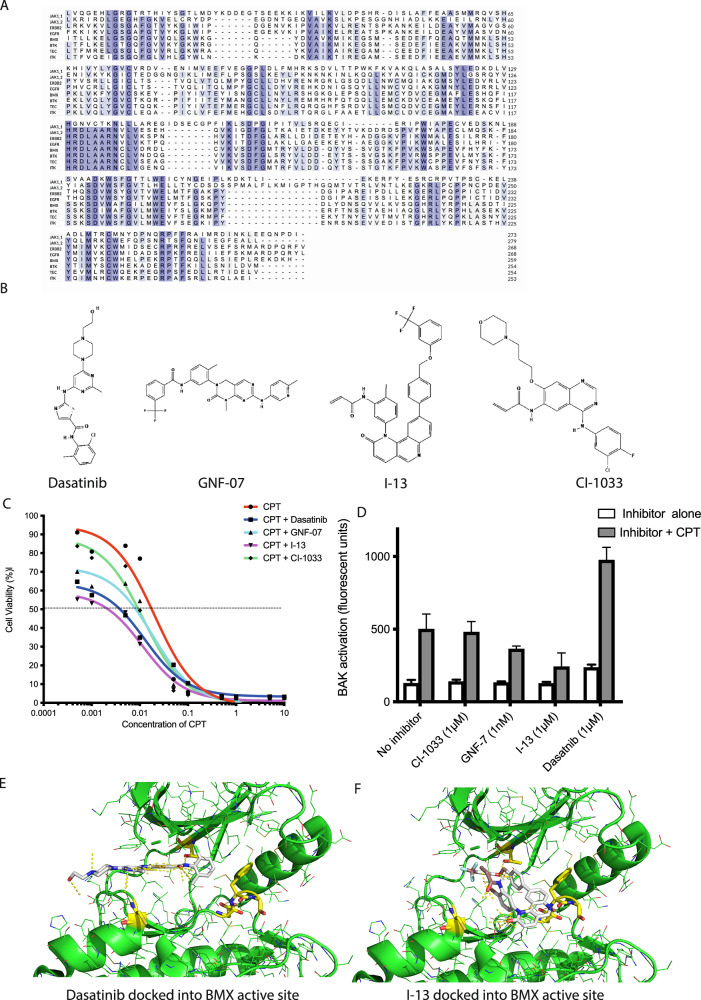
Characterisation of pan-tyrosine kinase inhibitors with activity against BMX. **A** Sequence alignment of kinase domain of BMX and closely related kinases, **B** Structures of tyrosine kinase inhibitors (TKIs) reported to inhibit BMX activity, **C** MTT assay to determine cell viability 96 h post concomitant treatment of HT1080 cells with Camptothecin (CPT) and TKIs, **D** Effect of concomitant treatment of HT1080 cells with Camptothecin (CPT) and TKIs on BAK activation via induction of N-terminal conformational change, **E** Crystal structure of Dasatinib bound to BMX (PDB:3SXR), **F** Docking of I-13 into BMX kinase domain (PDB:3SXR).

BMX kinase domain shares high sequence homology with the other TEC family members, and the closely related kinases JAK, ERBB2, and EGFR (Fig. 1A). Consequently, inhibitors designed to target these kinases also inhibit BMX. Inhibitors like Dasatinib, GNF-7, CL-1033, and I-13 have shown BMX inhibitory activity but were originally designed for other targets. For example Dasatinib, a BCR-ABL inhibitor, has a reported IC_50_ for its intended target of less than 1 nM and 10-fold higher IC_50_ for BMX. GNF-7, another BCR-ABL inhibitor, the EGFR inhibitor CL-1033, and I-13 also have reported activity against BMX [20] (Fig. 1B). We previously found that knockdown of BMX sensitized cancer cells to cell death through BAK activation [2], so we examined whether BMX inhibitors could phenocopy this sensitization to chemotherapeutic agents. Treating cells with a fixed dose of the tyrosine kinase inhibitor (TKIs) in combination with the chemotherapeutic agent camptothecin, known to induce apoptotic cell death via BAK activation [15], showed that all the TKIs increased the level of cell killing (Fig. 1C). However, only Dasatinib achieved this by increased BAK activation (Fig. 1D). Focusing on the two molecules which increased cell killing the most Dasatinib, which was sensitising cells to death via BAK activation and I-13, which was not, we investigated the interaction between these molecules and the BMX ATP binding pocket. In the published crystal structure of Dasatinib with the BMX kinase domain (PDB:3SXR), interactions are made with the front pocket cysteine C496 (coloured in yellow Fig. 1E) and the gatekeeper residue threonine 489 on the roof of the ATP binding pocket. Whereas docking of I-13 into the same structure is not predicted to interact with the front pocket cysteine, instead making extensive interactions with both the roof of the ATP binding pocket and the DFG motif, which in this structure is in the inactive out conformation (Fig. 1F).

**Fig. 2:**
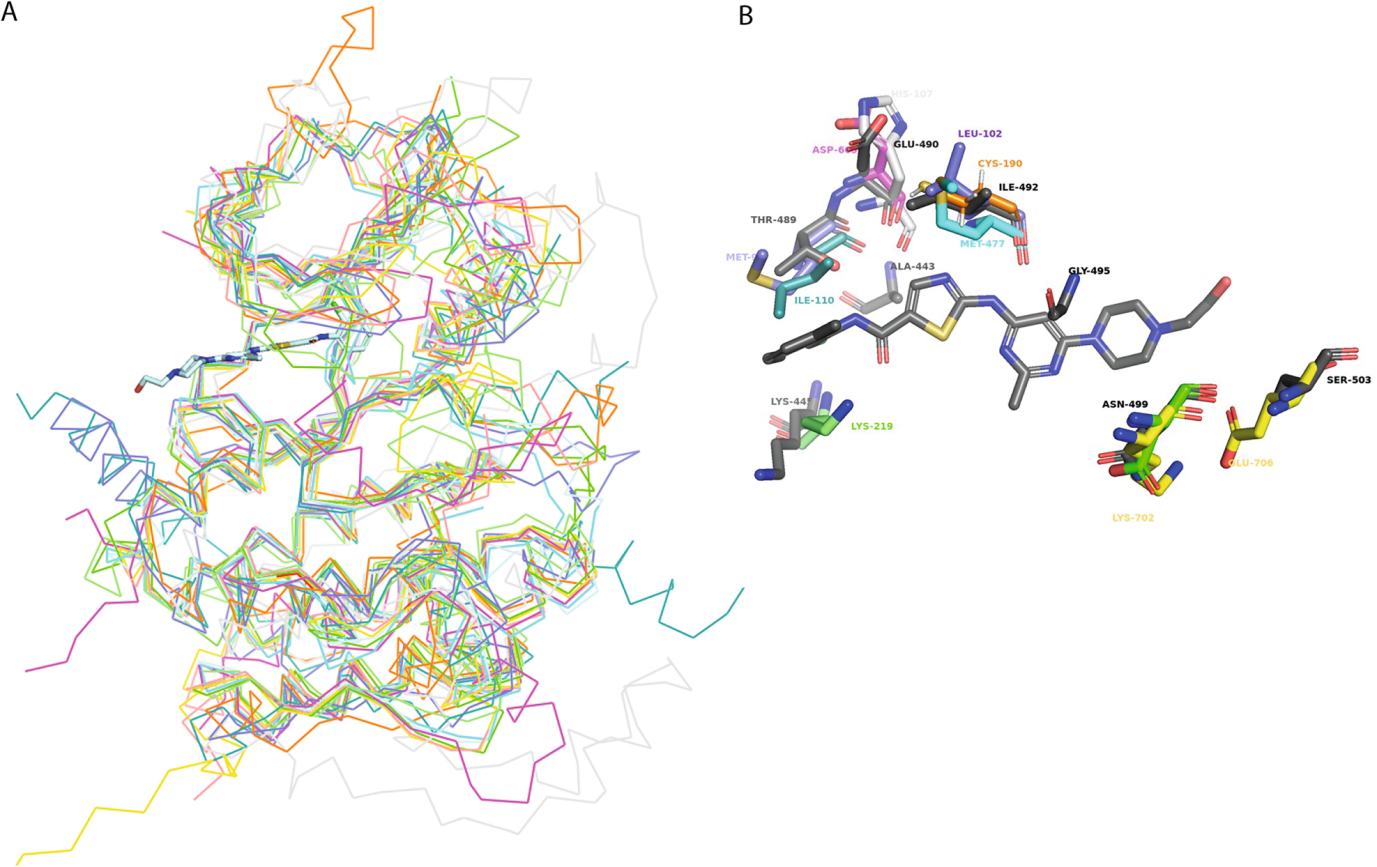
Comparison of kinase domain interactions with Dasatinib. **A** Overlay of crystal structures of human proteins that are bound to Dastanib (**B**) Dastanib variations of interacting residues of BMX (3SXR), BTK (3OCT), ABL (2GQG), STK24 (4QMS), MAPK (3LFA), DDR1 (6BSD), EPHA2 (5I9Y), MYT1 (5VCV).

Dasatinib has been extensively studied for its broad inhibitory activity across various kinases, including those in the SRC kinase family and ABL kinase family. Consequently, there are 27 deposited crystallographic structures of protein-Dasatinib complexes, 11 of which are human proteins. An overlay of the 11 human kinase structures bound to Dasatinib (Fig. 2A), revealed a consistent overall architecture among these proteins and indicates that Dasatinib binds to several conserved residues within the kinase domains. Notably, residues Ala443, Lys445, Thr489, Glu490, and Ile492 (numbering based on the BMX kinase) are among those conserved interactions. Conversely, Fig. 2B highlights the variability in certain residues across the kinase domains, underscoring the structural diversity within the kinase family despite the presence of conserved Dasatinib-binding residues. This structural variation may contribute to differences in binding affinity and specificity among different kinases, however, the highly conserved nature of protein kinase domains means developing highly specific inhibitors remains challenging.

**Fig. 3:**
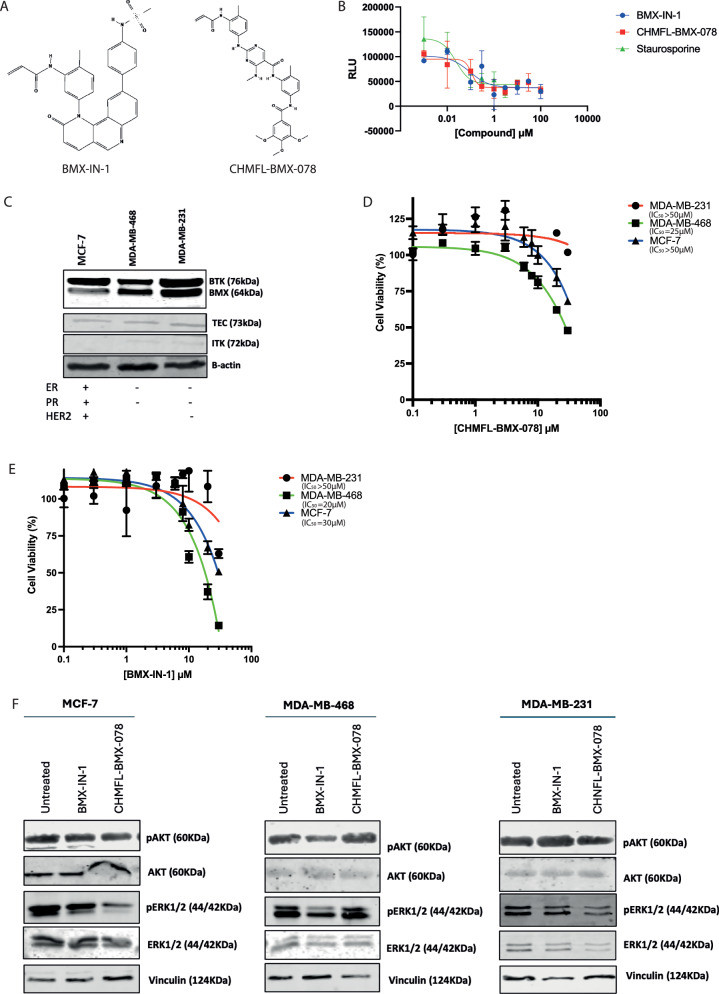
Characterisation of BMX-specific inhibitors in breast cancer cells. **A** Structures of BMX-specific inhibitors. **B** In vitro kinase assay determination of activity of inhibitors against purified full-length BMX protein. **C** Western blot analysis of BMX and BTK expression in 3 Breast Cancer cell lines, **D** Cell-titre Glo assay to determine effect of treatment with CHMFL-BMX-078 in Breast Cancer Cell lines (*n* = 3 +/− SEM), **E** Cell-titre Glo assay to determine effect of treatment with BMX-IN-1 in Breast Cancer Cell lines (*n* = 3 +/− SEM). Cell-titre Glo assays were performed 96 h post-treatment and are normalised relative to untreated controls. **F** Western blot analysis of the effect of 24 h BMX-IN-1 and CHMFL-BMX-078 treatment at 1xIC_50_ on downstream substrates in MCF-7, MDA-MB-468, and MDA-MB-231 breast cancer cells.

As Dasatinib is a broad pan-kinase inhibitor, to better evaluate the effect of BMX inhibition, we focused on molecules specifically designed to inhibit BMX. Two molecules from the published literature were selected BMX-IN-1 [21] and CHMFL-BMX-078 [22]. Structurally, these molecules are quite distinct (Fig. 3A). When tested in vitro against purified recombinant full-length protein, both inhibitors inhibited the kinase activity of BMX with similar IC_50_ values (Fig. 3B), as could the positive control non-selective kinase inhibitor staurosporine. To characterise these compounds in cell line models, we used 3 breast cancer cell lines, which had similar levels of TEC but different levels of the other family members. Of interest to this study was the varying levels of BMX in the different cell lines (MCF-7 low, MDA-MB-468 medium and MDA-MB-231 high; Fig. 3C). Determination of the cellular IC_50_ values for each of the compounds revealed the MDA-MB-231 cells with the high BMX levels had the highest IC_50_ and the MDA-MB-468 cells with a lower level of BMX expression were the most sensitive to the drugs. Cells treated with the IC_50_ dose of the drugs were effective at inhibiting BMX activity as measured by the modulation of downstream BMX targets, ERK1/2, and AKT. In the MDA-MB-468 cells, treatment with BMX-IN-1 caused decreased AKT phosphorylation, whereas CHMFL-BMX-078 resulted in decreased ERK1/2 phosphorylation in MCF-7 and MDA-MB-231 cells (Fig. 3F and quantified in Supplementary Fig. 1). These data suggest that these two compounds may be impacting BMX activity in different ways. Therefore, we focused on their specific mechanisms of BMX binding.

**Fig. 4:**
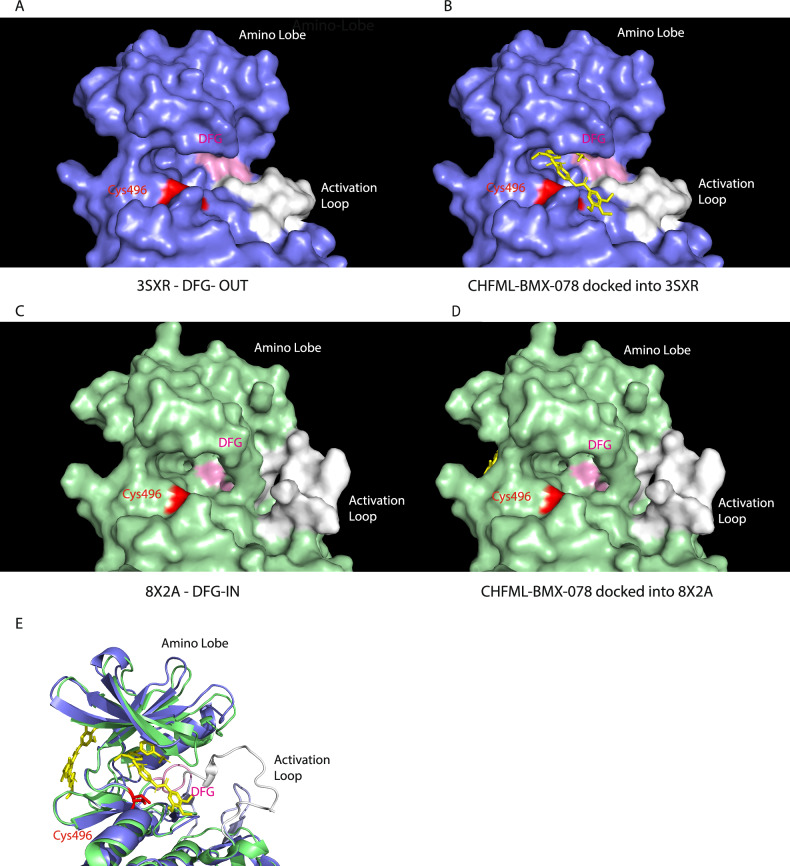
Position of DFG and activation loops in the BMX kinase domain affect CHMFL-BMX-078 binding. **A** Position of DFG and activation loop in DFG-out conformation in crystal structure PDB:3SXR and **B** docking of CHMFL-BMX-078, **C** Position of DFG and activation loop in DFG-in conformation in crystal structure PDB:8X2A and **D** docking of CHMFL-BMX-078, **E** Overlay of two crystal structures and relative position of CHMFL-BMX-078 docked into each structure.

**Fig. 5:**
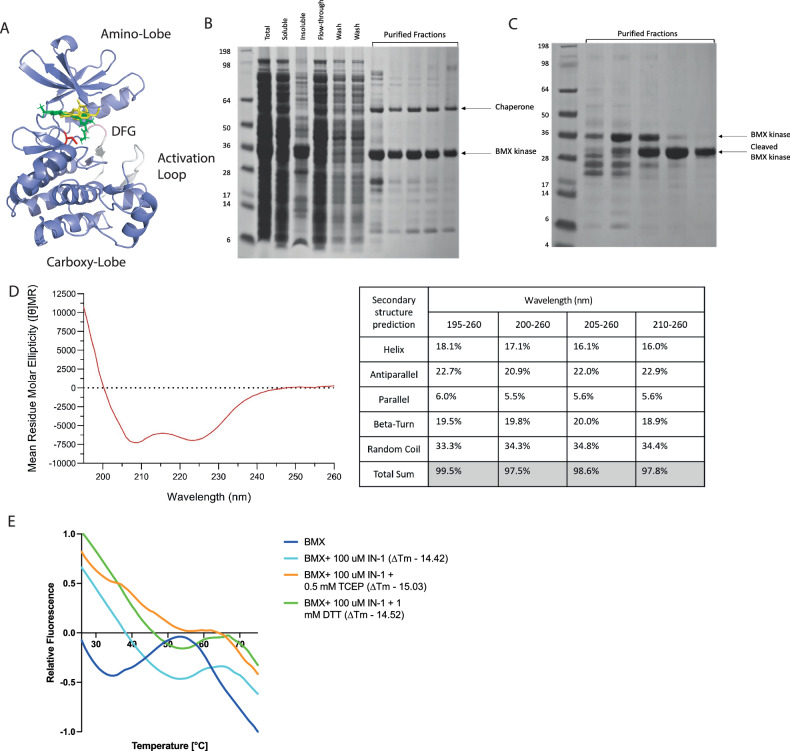
Reducing environment prevents BMX-IN-1 binding. **A** Structure of BMX kinase domain, **B** Analysis by SDS-PAGE of the expression and purification of HIS-tagged BMX-kinase domain via HIS-TRAP purified fractions correspond to the A280 peak observed from the column, **C** TEV cleavage and further purification by size exclusion chromatography to isolate cleaved kinase domain, **D** Circular Dichroism spectra of 0.1 mg/mL wild type BMX kinase domain in the Far-UV range (195–260 nm) at pH 7.4 and relative changes in BMX kinase domain secondary structure characteristics. CD performed at 20.42 °C for a pathlength of 10 mm. **E** Normalised thermal shift against 10 μM BMX kinase domain in response to BMX-IN-1 at 100 μM plus 0.5 mM TCEP or 1 mM DTT.

There are no published experimental structures of either of these inhibitors bound to BMX, so we performed docking of each of these inhibitors into published BMX kinase domain structures [3]. Firstly, CHMFL-BMX-078 was docked into the PDB structure 3SXR (BMX kinase domain in complex with Dasatinib). In this structure 3 amino acids have been mutated to allow crystallisation, and consequently the protein is found in the DFG ‘out’ inactive conformation with the activation loop in an open extended position (Fig. 4A). CHMFL-BMX-078 docks into the ATP binding pocket making contact with an additional allosteric site which includes the gate keeper Thr489 residue at the top of the ATP binding pocket (Fig. 4B). However, when the docking was repeated with the wild-type BMX kinase domain structure (PDB:8X2A), which is in the DFG ‘in’ active conformation (Fig. 4C), the inhibitor no longer bound into the ATP pocket (Fig. 4D). This confirms CHMFL-BMX-078 is a type II inhibitor and therefore likely more selective than other BMX inhibitors due to the additional interactions made within the ATP pocket. BMX-IN-1, however, is reported to be a Type I inhibitor which covalently modifies Cys496 at the front of the ATP pocket, and as such, binding of the compound would be unaffected by the position of the DFG and activation loops [23]. Docking of BMX-IN-1 into the kinase domain of BMX (PDB:3SXR) places the compound in the ATP pocket of BMX in the same position as was determined experimentally for its more potent analogue JS24 ([18]; Fig. 5A). Many tyrosine kinase inhibitors have been developed to target the cysteine residues due to their accessible location at the front of the ATP pocket and their nucleophilic properties. However, the ability of such residues to be covalently modified by inhibitors is dependent on the pH and chemical environment in which they are [24]. The cytoplasm of cells is known to be a highly reducing environment [25]; therefore, to characterise the effect of this environment on the activity of BMX-IN-1 activity we expressed wild-type BMX kinase domain (Fig. 5B, C). This yielded high purity protein, the secondary structure of which was confirmed to be correctly folded by CD spectroscopy in the far-UV wavelength range (195–260 nm; Fig. 5D). The secondary structure composition of the kinase domain was estimated to be 16% α-helix, 28.5% β-helix (22.9% antiparallel and 5.6% parallel), 18.9% β-turn and 34.4% random coils at 210–260 nm (Fig. 5D). To assess the effect of different conditions on the ability of BMX-IN-1 to interact with the kinase domain we used Differential Scanning Fluorimetry (DSF) to assess the concentration of BMX-IN-1 required to increase the thermal stability of the BMX kinase domain. Analysis was carried out both at room temperature to assess ligand binding after a prolonged period of saturation with the protein to ensure full or near-full binding site occupation, and following a 2 h incubation at 4° C to assess binding without saturation, therefore showing the affinity of the inhibitors. A lack of difference between these two sets of results suggested that BMX-IN-1 has a high affinity for the BMX kinase domain and rapidly saturates the protein (Fig. 5E).

To determine the impact of a reducing environment on the effectiveness of these BMX-IN-1, the DSF analysis was repeated in the presence of 500 μM TCEP or 1 mM DTT, which would reduce the cys496 residue. TCEP is a stronger reducing agent than DTT, allowing for a comparison of binding in different reducing environments. Without incubation, BMX-IN-1 caused a noticeable and significant increase in melting temperature from a peak at 55 °C to a peak at 65 °C (Fig. 5E). In a strongly reducing environment in the presence of TCEP there was a significant shift in melting temperature curve in response to BMX-IN-1 (Fig. 5E). Conversely, in a less reducing environment generated by the presence of 1 mM of DTT, we did not observe a significant change in melting temperature (Fig. 5E). Interestingly, these curves did not follow the expected patterns of rightward shift, rather showing dramatic increases in fluorescence. Without incubation at 4 °C, TCEP caused a −242.36 reduction in fluorescence past 62 °C (Fig. 5E). For DTT after 2 h of incubation of 4 °C, the curve experienced a 22.84 fold increase in fluorescence past 60 °C (Supplementary Fig. 2 and Supplementary Table 1). This suggests the reducing agents did not affect the thermal stability of the ligand-protein complex, but rather the binding event.

**Fig. 6:**
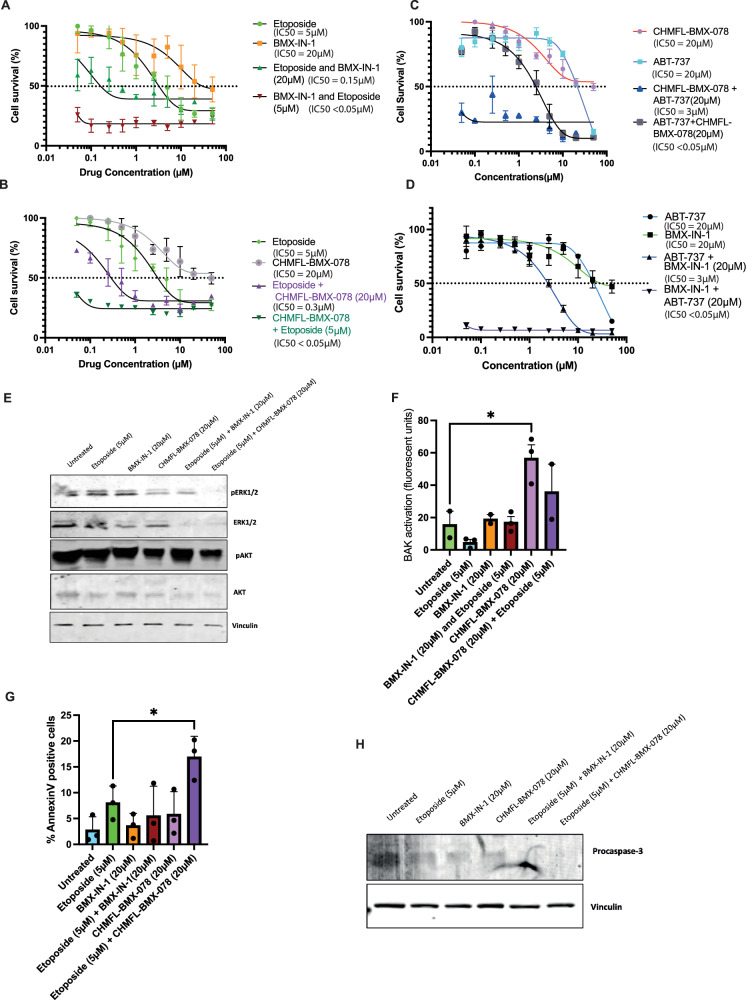
Combination of BMX-specific inhibitors with apoptosis-inducing agents sensitises MDA-MB-231 cells to death. **A** MTT assay to determine the effect of BMX-IN-1 and Etoposide alone and in combination. **B** MTT assay to determine the effect of CHMFL-BMX-078 and Etoposide alone and in combination. **C** MTT assay to determine the effect of CHMFL-BMX-078 and ABT-737 alone and in combination. **D** MTT assay to determine the effect of BMX-IN-1 and ABT-737 alone and in combination. For all MTT assays were conducted 96 h post treatment (*n* = 3 +/− SEM). **E** Western blot analysis of the effect of 24 h BMX-IN-1 (20 µM) or CHMFL-BMX-078 (20 µM) treatment in combination with Etoposide (5 µM) on downstream substrate ERK and AKT phosphorylation. **F** Effect of treatment of BMX inhibitors alone (BMX-IN-1 (20 µM) and ABT-737 (20 µM)) or in combination with etoposide (5 µM) on BAK activation via induction of N-terminal conformational change (*n* = 3 +/− SEM *P < 0.05). Effect of treatment of BMX inhibitors alone (BMX-IN-1 (20 µM) and ABT-737 (20 µM)) or in combination with etoposide (5 µM) on apoptosis induction via (**G**) annexin-V staining (*n* = 3 +/− SEM *P < 0.05) and (**H**) western blot analysis of Procaspase-3 cleavage.

As these two inhibitors work via two different mechanisms, CHMFL-BMX-078 is dependent on the DFG conformation of BMX, and BMX-IN-1 is dependent on the chemical environment the cys496 is in, we next evaluated if these BMX inhibitors could sensitise cells to cell death in combination with other chemotherapeutic and targeted agents. Utilising the MBA-MD-231 breast cancer cell line, which was the most resistant to treatment with BMX inhibitors alone, we determined the cellular IC_50_ values for each compound. In this cell line, BMX-IN-1 was marginally more potent compared to CHMFL-BMX-078, but both compounds had IC_50_ values in the micromolar range (Fig. 6A, B). The cells were treated with either a fixed dose of the BMX inhibitors in combination with variable doses of the chemotherapeutic agent etoposide, known to induce cell death via BAK activation. Or a fixed dose of Etoposide with variable doses of the BMX inhibitors (Fig. 6A, B). We observed that both compounds could increase the level of cell killing (Fig. 6A, B), with CHMFL-BMX-078 causing a significant decrease in the IC_50_ by 10-fold to 0.3 µM (*P* = 0.0178), whereas BMX-IN-1 decreased the IC_50_ 20-fold to 0.15 µM (*P* = 0.016). Sensitisation was also observed when either BMX-IN-1 or CHMFL-BMX-078 was combined with the targeted BH3-mimetic ABT-737 (8-fold and 6-fold decrease in IC_50_, respectively; *P* < 0.01), which specifically induces BCL-2 family-driven apoptosis in cells (Fig. 6C, D). We observed an even greater sensitisation when a fix dose of the chemotherapeutic was combined with variable doses of the BMX inhibitors (Fig. 6A–D). Further analysis of these drug combinations using Combusyn Analysis Software that uses the median-effect equation to determine the combination index (CI) for drug interactions [26] confirmed that with optimal dose selection synergistic outcomes can be achieved for all the drug combinations tested in this study (Supplementary Fig. 3). Analysis of ERK and AKT phosphorylation as downstream substrates of BMX in these cells following treatment revealed that in this cell line CHMFL-BMX-078 either alone or in combination with etoposide caused a decrease in both ERK and AKT phosphorylation, suggesting at this dose BMX kinase activity was being modulated. Whereas, only when BMX-IN-1 was used in combination were decreases in ERK observed (Fig. 6D and quantified in Supplementary Fig. 4). We have previously reported that BAK is also a BMX substrate, and BAK activation can be used as a surrogate marker for BAK dephosphorylation [15]. Treatment of MBA-MD-231 cells with CHMFL-BMX-078, both alone and in combination, resulted in significantly increased BAK activation compared to untreated controls 2 h post-treatment (Fig. 6E), as the BMX inhibitor directly modulates BAK phosphorylation status and ability to be activated. Etoposide, however, induces DNA double-strand breaks, which in turn induce mitochondrial apoptosis, so BAK activation may occur at later time points. Additionally, cells treated with the combination of CHMFL-BMX-078 and Etoposide resulted in a significant increase in apoptotic cells compared to either drug alone as determine by Annexin-V staining (Fig. 6F) However, treatment with BMX-IN-1 both alone and in combination did not increase BAK activation or annexin V positive cells compared to etoposide alone, suggesting the cell killing observed by this drug was not via apoptosis.

## Discussion

Overcoming resistance in cancer therapies requires novel strategies, and while BMX has been identified as a promising target, no BMX inhibitors have advanced to clinical trials. This study evaluates commercially available BMX inhibitors to understand their mechanisms of action and assess whether targeting BMX, either alone or in combination with existing treatments, could improve cancer treatment outcomes.

A significant challenge in targeting kinases is the sequence conservation across related kinases, making inhibitors less specific, particularly type I inhibitors, like Dasatinib and BMX-IN-1, which demonstrate low specificity for BMX, and while pan-kinase inhibitors have benefits [27], they also carry a risk of toxicity due to off-target effects. For example, many of the BTK inhibitors currently in clinical trials are reporting dose-limiting toxicities of thrombocytopenia and neutropina [28] in addition to dermatological toxicities [29], all of which are limiting their clinical uses. Thus, BMX inhibitors that also target BTK may share these toxicities [18].

The BMX-specific inhibitors, BMX-IN-1 and CHMFL-BMX-078, show similar inhibitory activity on purified protein and in cells when used as single agents, but differ in their effects on downstream pathways. BMX-IN-1 primarily affects AKT signalling, while CHMFL-BMX-078 impacts ERK1/2 signalling (Fig. 3). These differences could arise from their distinct mechanisms: BMX-IN-1 is a type I inhibitor that targets the NCap Cysteine residue covalently interacting with it to prevent ATP binding [24], while CHMFL-BMX-078 is a type II inhibitor that our modelling confirms only binds when BMX is in the inactive DFG-out conformation [30] (Fig. 4) and the activation loop is extended [22]. The additional allosteric contacts CHMFL-BMX-078 makes within the ATP binding pocket, increases the specificity of type II inhibitors for BMX. However, the amount of BMX that adopt the DFG-out conformation in cancer cells requires further investigation to determine if this is a viable biomarker for patient selection.

The cysteine residue at the front of the ATP-binding pocket has emerged as one of the most targeted amino acids for the development of kinase inhibitors [31] due to its high nucleophilicity. We found that the reactivity of the cys496 residue in BMX-IN-1 is highly dependent on the cellular redox environment. In cancer, the cellular redox state is often altered and shifted towards a reducing environment, as this gives the cancer cells a survival advantage promoting proliferation and the survival of transformed cells [25]. One direct consequence of this change in chemical environment is a decrease in the efficacy of chemotherapeutic agents. Our data show that reducing agents like TCEP and DTT decrease the binding of BMX-IN-1 to BMX, suggesting that the redox state may contribute to drug resistance in cancers [25]. This underscores the challenge of developing effective kinase inhibitors in the context of altered cellular environments.

Our hypothesis that inhibiting BMX would be most effective in combination with other chemotherapeutic agents [2] is supported by our data showing that both BMX-IN-1 and CHMFL-BMX-078 increase cell death when combined with chemotherapeutic agents like etoposide (Fig. 6A, B). Analysis of the mechanism of cell death indicated that only CHMFL-BMX-078 causes cell death via apoptosis, BMX-IN-1 however, induces cell death by an alternate currently undetermined mechanism. These differences may be due to the inhibitors’ distinct effects on downstream signalling—CHMFL-BMX-078 modulates ERK1/2 and BAK activation, whereas BMX-IN-1 primarily affects AKT signalling. Both BMX-IN-1 and CHMFL-BMX-078 have previously been reported to increase cell killing in combination with AKT inhibitors [21, 32, 33] compared to either agent alone, suggesting that inhibition of BMX alone does not sufficiently modulate the AKT signalling pathway to produce an effect. This highlights that determining the mechanism by which novel BMX inhibitors cause increased cell killing will require careful analysis both of cell death mechanism and the downstream signalling outcomes.

In conclusion, BMX inhibitors are most effective in combination therapies, but the need for more selective inhibitors remains. Our findings highlight the importance of understanding the mechanisms behind BMX inhibitor-induced cell death and suggest that careful analysis of both signalling and apoptosis pathways is crucial in developing effective BMX-targeted therapies.

**Supplementary Materials:** The following supporting information can be downloaded at: www.mdpi.com/xxx/s1, Figure S1: Quantification of change in downstream substrates. Figure S2: Reducing agent DTT did not affect the thermal stability of the BMX-IN-1-protein complex. Figure S3: Determination of combination index for Etoposide & CHMFL-BMX-078 and ABT737 and CHMFL-BMX-078. Table S1: Comparison of effect on melting temperature following room temperature or 4 °C incubation.

## Supplementary information

Supplementary Data and Methods

Raw western blot files

Checklist

## Data Availability

All data included within the manuscript are accessible via the Leicester Research Archive. The re-analysis of existing data, which are openly available at Protein DataBank (https://www.rcsb.org/) locations cited in the ‘References’ section.’

## References

[1] Hanahan D. Hallmarks of Cancer: New Dimensions. Cancer Discov. 2022;12:31–46. 35022204 10.1158/2159-8290.CD-21-1059

[2] Fox JL, Storey A. BMX Negatively Regulates BAK Function, Thereby Increasing Apoptotic Resistance to Chemotherapeutic Drugs. Cancer Res. 2015;75:1345–55. 25649765 10.1158/0008-5472.CAN-14-1340 PMC4384990

[3] Muckelbauer J, Sack JS, Ahmed N, Burke J, Chang CY, Gao M, et al. X-ray crystal structure of bone marrow kinase in the x chromosome: a Tec family kinase. Chem Biol Drug Des. 2011;78:739–48. 21883956 10.1111/j.1747-0285.2011.01230.x

[4] Chen S, Cai C, Sowalsky AG, Ye H, Ma F, Yuan X, et al. BMX-Mediated Regulation of Multiple Tyrosine Kinases Contributes to Castration Resistance in Prostate Cancer. Cancer Res. 2018;78:5203–15. 30012673 10.1158/0008-5472.CAN-17-3615 PMC6139052

[5] Cenni B, Gutmann S, Gottar-Guillier M. BMX and its role in inflammation, cardiovascular disease, and cancer. Int Rev Immunol. 2012;31:166–73. 22449076 10.3109/08830185.2012.663838

[6] Mano H. Tec family of protein-tyrosine kinases: an overview of their structure and function. Cytokine Growth Factor Rev. 1999;10:267–80. 10647781 10.1016/s1359-6101(99)00019-2

[7] Vargas L, Hamasy A, Nore BF, Smith CI. Inhibitors of BTK and ITK: state of the new drugs for cancer, autoimmunity and inflammatory diseases. Scand J Immunol. 2013;78:130–9. 23672610 10.1111/sji.12069

[8] Wang X, Kokabee L, Kokabee M, Conklin DS. Bruton’s Tyrosine Kinase and Its Isoforms in Cancer. Front Cell Dev Biol. 2021;9:668996. 34307353 10.3389/fcell.2021.668996 PMC8297165

[9] Tam C, Thompson PA. BTK inhibitors in CLL: second-generation drugs and beyond. Blood Adv. 2024;8:2300–09. 38478390 10.1182/bloodadvances.2023012221 PMC11117011

[10] Weeks S, Harris R, Karimi M. Targeting ITK signaling for T cell-mediated diseases. iScience. 2021;24:102842. 34368657 10.1016/j.isci.2021.102842 PMC8326193

[11] Li K, Pan WT, Ma YB, Xu XL, Gao Y, He YQ, et al. BMX activates Wnt/β-catenin signaling pathway to promote cell proliferation and migration in breast cancer. Breast Cancer. 2020;27:363–71. 31728872 10.1007/s12282-019-01024-8

[12] Eldeeb MA, Fahlman RP. Phosphorylation Impacts N-end Rule Degradation of the Proteolytically Activated Form of BMX Kinase. J Biol Chem. 2016;291:22757–68. 27601470 10.1074/jbc.M116.737387 PMC5077209

[13] Chen S, Jiang X, Gewinner CA, Asara JM, Simon NI, Cai C, et al. Tyrosine kinase BMX phosphorylates phosphotyrosine-primed motif mediating the activation of multiple receptor tyrosine kinases. Sci Signal. 2013;6:ra40. 23716717 10.1126/scisignal.2003936 PMC3735445

[14] Jiang T, Guo Z, Dai B, Kang M, Ann DK, Kung HJ, et al. Bi-directional regulation between tyrosine kinase Etk/BMX and tumor suppressor p53 in response to DNA damage. J Biol Chem. 2004;279:50181–9. 15355990 10.1074/jbc.M409108200

[15] Fox JL, Ismail F, Azad A, Ternette N, Leverrier S, Edelmann MJ, et al. Tyrosine dephosphorylation is required for Bak activation in apoptosis. EMBO J. 2010;29:3853–68. 20959805 10.1038/emboj.2010.244 PMC2989102

[16] Fox JL. Cancer chemoresistance and BAK. Oncoscience. 2015;2:932–3. 26909357 10.18632/oncoscience.276 PMC4741396

[17] Rajantie I, Ekman N, Iljin K, Arighi E, Gunji Y, Kaukonen J, et al. Bmx tyrosine kinase has a redundant function downstream of angiopoietin and vascular endothelial growth factor receptors in arterial endothelium. Mol Cell Biol. 2001;21:4647–55. 11416142 10.1128/MCB.21.14.4647-4655.2001 PMC87133

[18] Seixas JD, Sousa BB, Marques MC, Guerreiro A, Traquete R, Rodrigues T, et al. Structural and biophysical insights into the mode of covalent binding of rationally designed potent BMX inhibitors. RSC Chem Biol. 2020;1:251–62. 34458764 10.1039/d0cb00033g PMC8341910

[19] Eberhardt J, Santos-Martins D, Tillack AF, Forli S. AutoDock Vina 1.2.0: New Docking Methods, Expanded Force Field, and Python Bindings. J Chem Inf Model. 2021;61:3891–98. 34278794 10.1021/acs.jcim.1c00203 PMC10683950

[20] Jarboe JS, Dutta S, Velu SE, Willey CD. Mini-review: bmx kinase inhibitors for cancer therapy. Recent Pat Anticancer Drug Discov. 2013;8:228–38. 23198769 10.2174/15748928113089990043

[21] Liu F, Zhang X, Weisberg E, Chen S, Hur W, Wu H, et al. Discovery of a selective irreversible BMX inhibitor for prostate cancer. ACS Chem Biol. 2013;8:1423–8. 23594111 10.1021/cb4000629

[22] Liang X, Lv F, Wang B, Yu K, Wu H, Qi Z, et al. Discovery of 2-((3-Acrylamido-4-methylphenyl)amino)-N-(2-methyl-5-(3,4,5-trimethoxybenzamido)p henyl)-4-(methylamino)pyrimidine-5-carboxamide (CHMFL-BMX-078) as a Highly Potent and Selective Type II Irreversible Bone Marrow Kinase in the X Chromosome (BMX) Kinase Inhibitor. J. Med Chem. 2017;60:1793–816. 10.1021/acs.jmedchem.6b01413 28140585

[23] Treiber DK, Shah NP. Ins and outs of kinase DFG motifs. Chem Biol. 2013;20:745–6. 23790484 10.1016/j.chembiol.2013.06.001

[24] Liu R, Zhan S, Che Y, Shen J. Reactivities of the Front Pocket N-Terminal Cap Cysteines in Human Kinases. J Med Chem. 2022;65:1525–35. 34647463 10.1021/acs.jmedchem.1c01186 PMC8812259

[25] Chun KS, Kim DH, Surh YJ. Role of Reductive versus Oxidative Stress in Tumor Progression and Anticancer Drug Resistance. Cells. 2021;30:10. 10.3390/cells10040758 PMC8065762 33808242

[26] Chou TC. Theoretical basis, experimental design, and computerized simulation of synergism and antagonism in drug combination studies. Pharmacol Rev. 2006;58:621–81. 16968952 10.1124/pr.58.3.10

[27] Montoya S, Soong D, Nguyen N, Affer M, Munamarty SP, Taylor J. Targeted Therapies in Cancer: To Be or Not to Be, Selective. Biomedicines. 2021;01:9. 10.3390/biomedicines9111591 PMC8615814 34829820

[28] Aalipour A, Advani RH. Bruton’s tyrosine kinase inhibitors and their clinical potential in the treatment of B-cell malignancies: focus on ibrutinib. Ther Adv Hematol. 2014;5:121–33. 25360238 10.1177/2040620714539906 PMC4212313

[29] Sibaud V, Beylot-Barry M, Protin C, Vigarios E, Recher C, Ysebaert L. Dermatological Toxicities of Bruton’s Tyrosine Kinase Inhibitors. Am J Clin Dermatol. 2020;21:799–812. 32613545 10.1007/s40257-020-00535-x

[30] Ung PM, Schlessinger A. DFGmodel: predicting protein kinase structures in inactive states for structure-based discovery of type-II inhibitors. ACS Chem Biol. 2015;10:269–78. 25420233 10.1021/cb500696t PMC4301084

[31] Huang F, Han X, Xiao X, Zhou J. Covalent Warheads Targeting Cysteine Residue: The Promising Approach in Drug Development. Molecules. 2022;10:27. 10.3390/molecules27227728 PMC9694382 36431829

[32] Jiang S, Jiang T, Huang H, Chen X, Li L, Wang Z, et al. CHMFL-BMX-078, a BMX inhibitor, overcomes the resistance of melanoma to vemurafenib via inhibiting AKT pathway. Chem Biol Interact. 2022;351:109747. 34813779 10.1016/j.cbi.2021.109747

[33] Griffiths GJ, Dubrez L, Morgan CP, Jones NA, Whitehouse J, Corfe BM, et al. Cell damage-induced conformational changes of the pro-apoptotic protein Bak in vivo precede the onset of apoptosis. Journal cell Biol. 1999;144:903–14. 10085290 10.1083/jcb.144.5.903 PMC2148192

