# Supplementary material for: Inhibition of tyrosine kinase BMX increases cell death in response to existing chemotherapeutic agents overcoming apoptotic resistance in cancer
Source: Cell Death Dis. 2025 Nov 10;16(1):813. doi: 10.1038/s41419-025-08131-9 (PMC12603249; doi:10.1038/s41419-025-08131-9)
Supplement: Supplementary file 1 — Supplementary Data and Methods [file 41419_2025_8131_MOESM1_ESM.pdf]

Supplementary Data

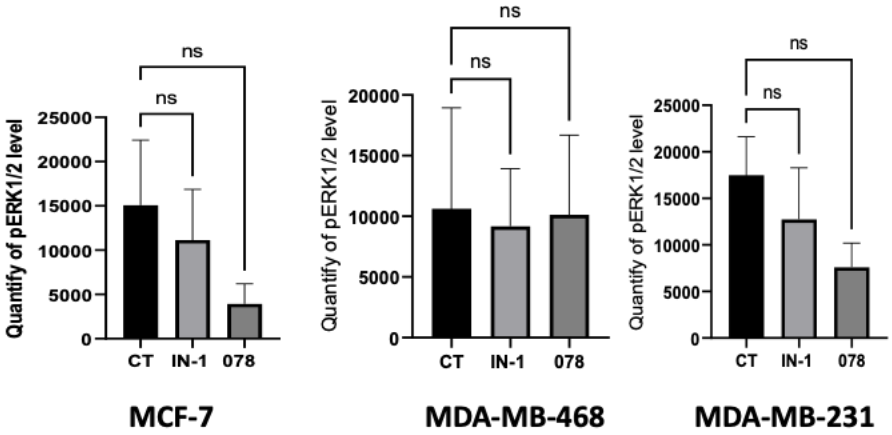

Supplementary Figure 1: Quantification of change in ERK1/2 phosphorylation in western blots. The density profile of each pERK1/2 band in three biological repeats was determined using ImageJ. Mean density in each condition are presented ± SEM.

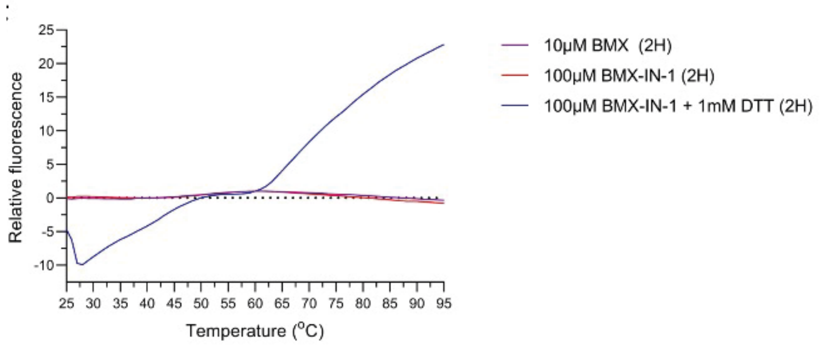

Supplementary Figure 2: Reducing agent DTT did not affect the thermal stability of the BMX-IN-1-protein complex. Melting temperature change over 10 µM BMX kinase domain in response to 100 µM BMX-IN-1 at room temperature and following 2H incubation at 4℃, without reducing agent and in the presence of 1 mM DTT. Temperature ranged from 25℃ to 85℃, increasing at increments of 0.06℃/s.

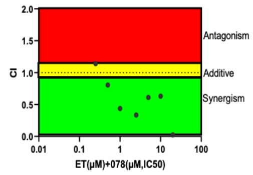

Data for Non-Constant Combo: (ET+078)

ET	078	Effect	CI
50.0	20.0	0.65	4.69912
20.0	20.0	0.98	0.02415
10.0	20.0	0.75	0.63144
5.0	20.0	0.69	0.61130
2.5	20.0	0.73	0.33565
1.0	20.0	0.65	0.43833
0.5	20.0	0.53	0.80544
0.25	20.0	0.46	1.14038
0.1	20.0	0.33	2.42314
0.05	20.0	0.27	3.61832

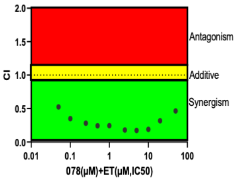

CI Data for Non-Constant Combo: (ET+078)

ET	078	Effect	CI
5.0	50.0	0.76	0.46360
5.0	20.0	0.76	0.31801
5.0	10.0	0.8	0.18924
5.0	5.0	0.8	0.17250
5.0	2.5	0.79	0.17997
5.0	1.0	0.75	0.24486
5.0	0.5	0.75	0.24222
5.0	0.25	0.73	0.28148
5.0	0.1	0.7	0.34976
5.0	0.05	0.64	0.52433

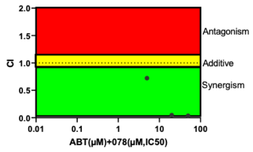

CI Data for Non-Constant Combo: µM (078 +abt)

Dose 078	Dose abt	Effect	CI
20.0	50.0	0.9	0.03646
20.0	20.0	0.88	0.04799
20.0	10.0	0.18	23.1340
20.0	5.0	0.54	0.72201
20.0	2.5	0.29	3.68974
20.0	1.0	0.21	6.55910
20.0	0.5	0.12	17.9647
20.0	0.25	0.04	130.704
20.0	0.1	0.05	68.6276
20.0	0.05	0.17	8.34504

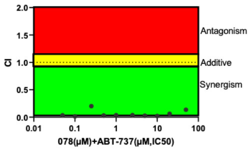

CI Data for Non-Constant Combo: 078abt (078 +abt)

Dose 078	Dose abt	Effect	CI
50.0	20.0	0.87	0.13506
20.0	20.0	0.86	0.06245
10.0	20.0	0.89	0.02120
5.0	20.0	0.82	0.02807
2.5	20.0	0.75	0.03737
1.0	20.0	0.72	0.03577
0.5	20.0	0.72	0.03045
0.25	20.0	0.56	0.20013
0.1	20.0	0.76	0.01454
0.05	20.0	0.7	0.03392

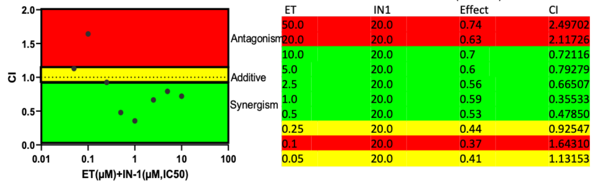

CI Data for Non-Constant Combo: (ET+IN-1)

ET	IN1	Effect	CI
50.0	20.0	0.74	2.49702
20.0	20.0	0.63	2.11726
10.0	20.0	0.7	0.72116
5.0	20.0	0.6	0.79279
2.5	20.0	0.56	0.66507
1.0	20.0	0.59	0.35533
0.5	20.0	0.53	0.47850
0.25	20.0	0.44	0.92547
0.1	20.0	0.37	1.64310
0.05	20.0	0.41	1.13153

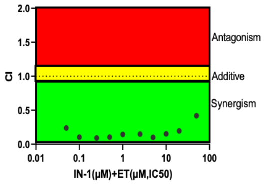

CI Data for Non-Constant Combo: (ET+IN1)

ET	IN1	Effect	CI
5.0	50.0	0.77	0.41845
5.0	20.0	0.81	0.19580
5.0	10.0	0.82	0.15208
5.0	5.0	0.85	0.10035
5.0	2.5	0.81	0.14837
5.0	1.0	0.81	0.14430
5.0	0.5	0.84	0.10459
5.0	0.25	0.85	0.09287
5.0	0.1	0.84	0.10386
5.0	0.05	0.75	0.23960

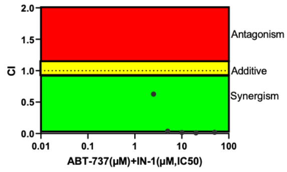

CI Data for Non-Constant Combo: abt (in-1+abt)

Dose in-1	Dose abt	Effect	CI
20.0	50.0	0.86	0.01937
20.0	20.0	0.87	0.01091
20.0	10.0	0.84	0.01645
20.0	5.0	0.78	0.03683
20.0	2.5	0.5	0.62761
20.0	1.0	0.28	5.00693
20.0	0.5	0.2	13.0826
20.0	0.25	0.14	33.5707
20.0	0.1	0.11	61.1432
20.0	0.05	0.1	77.0588

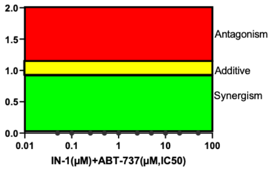

CI Data for Non-Constant Combo: in-1 (in-1+abt)

Dose in-1	Dose abt	Effect	CI
50.0	20.0	0.94	0.00318
20.0	20.0	0.95	9.22E-4
10.0	20.0	0.94	8.75E-4
5.0	20.0	0.94	5.87E-4
2.5	20.0	0.93	6.81E-4
1.0	20.0	0.92	8.20E-4
0.5	20.0	0.92	7.62E-4
0.25	20.0	0.93	4.93E-4
0.1	20.0	0.93	4.81E-4
0.05	20.0	0.9	0.00140

Supplementary Figure 3: Determination of Combination Index for CHMFL-BMX-078 and BMX-IN-1 in combination with either Etoposide or ABT737 in MDA-MBA-231 Drugs were dosed concomitantly with one drug at a fixed dose and the other drug varying. Data was analysed using Combusyn software.

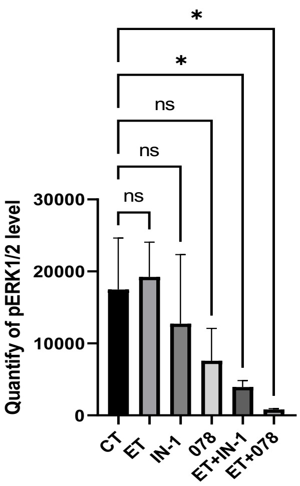

Supplementary Figure 4: Quantification of change in pERK1/2 following treatment with Etoposide alone or in combination with BMX-IN-1 or CHMFL-BMX-078. The density profile of each pERK1/2 band in three biological repeats was determined using ImageJ. Mean density in each condition are presented + SEM.

Compound	Tm (°C)	BMX Tm (°C)	ΔTm (°C)	BMX ΔTm (°C)	Δ/°C	BMX Δ/°C
**BMX-IN-1 (100μM) (RT)**	58.39	43.97	14.42	00.00	18.30	07.90
**BMX-IN-1 (100μM) (2H)**	52.00	49.51	02.49	00.00	07.90	06.90
**BMX-IN-1 (100μM) + TCEP (500μM) (RT)**	59.00	43.97	15.03	00.00	35.90	07.90
**BMX-IN-1 (100μM) + TCEP (500μM) (2H)**	55.25	49.51	05.74	00.00	17.70	06.90
**BMX-IN-1 (100μM) + DTT (1mM) (RT)**	58.49	43.97	14.52	00.00	20.90	07.90
**BMX-IN-1 (100μM) + DTT (1mM) (2H)**	N/A	49.51	N/A	00.00	N/A	06.90

Supplementary Table 1. Comparison of effect on melting temperature following room temperature or 4℃ incubation. Melting temperature change over 10 µM BMX kinase domain in response to 100 µM BMX-IN-1 at room temperature and following 2H incubation at 4℃, without reducing agent and in the presence of 500 µM TCEP or 1 mM DTT as determined by DSF. Temperature ranged from 25℃ to 85℃, increasing at increments of 0.06℃/s.

Supplementary Methods:

Flow cytometric analysis: Flow cytometric analysis of BAK conformation was determined using Ab-1 (AM03; Calbiochem) as previously described [15]. Briefly, after treatment cells were fixed in paraformaldehyde (0.25% PFA/PBS) for 10 min at room temperature. Cells were collected by scrapping and washed with PBS. Cells were permeabilised with 0.01% saponin/PBS and incubated with Bak Ab-1 primary antibody (AM03; Calbiochem) or mouse IgG1 (Pharmingen) for 30 min at 4°C. Cells were then washed and incubated with rabbit anti-mouse phycoerythrin or with goat anti-mouse Allophycocyanin secondary antibody for 30 min at 4°C. Cells were washed and resuspended in PBS for analysis with a Canto II analyser (Beckman). Approximately, 10 000 cells were analysed per sample. To quantify the flow cytometric results using Ab-1, the data were then manipulated as previously described [32]. Briefly, cells exhibiting a light scatter profile associated with apoptotic cells were gated out and the median Bak-associated fluorescence was determined by subtracting the median fluorescence of the parallel IgG control from each test sample. The median value was then multiplied by the percentage of Bak-positive cells as determined by the IgG control to give the Ab-1 Bak-specific fluorescence of each sample. Three biological repeats were performed per experiments and statistical differences determine by t-test.

Detection of Annexin V was used as a marker for apoptotic cells as described [1]. Briefly, cells were treated with BMX-IN-1 (20μM), CHMFL-BMX-078 (20μM) or ET (5μM) or a combination of the compounds for 24 hours. Cells were harvested and stained with AnnexinV-FITC in 10 mM HEPES buffer pH7.4, 140 mM $NACl_2$, 2.5 mM $CaCl_2$. Cells were resuspended in PBS for analysis using a Canto II analyser (Beckman). In all cases 10,000 cells were analyzed per

sample, three biological repeats were performed per experiments and statistical differences determine by t-test.

Western blotting: Cells were washed with PBS, collected by scrapping and lysed at 4°C for 1 h. Lysis buffer contained 50 mM Tris–HCl, 150 mM NaCl (pH 7.5), 2 mM EDTA (pH8.0), 1% CHAPS, Complete mini protease inhibitor tablet (Roche), 1% phosphatase inhibitor cocktail 1 (Sigma) and 1% phosphatase inhibitor 2 (Sigma). Lysates were centrifuged (15 000 r.p.m. for 15 min at 4°C) and protein quantification was carried out using Bradford protein assay (Perbio Science UK). The resulting protein extracts were then separated by SDS–polyacrylamide gel electrophoresis and electroblotted onto PVDF membranes. Antibodies used were as follows: anti-Btk (#3547; Cell Signaling Technology), anti-BMX (#610792; BD Bioscience), anti-pERK1/2 (#9101; Cell Signaling Technology), total anti-ERK1/2 (#9102; Cell Signaling Technology) anti-pAKT (#9271; Cell Signaling Technology), total anti-AKT (#9272; Cell Signaling Technology), anti-Vinculin (#VMA00895; BioRad). Secondary antibodies were goat anti-mouse or goat anti-rabbit IRDye fluorescently tagged (used at a dilution of 1:10,000; LiCor UK). Reactive proteins were visualised using Odessey (LiCor).

Protein expression: Wild-type BMX kinase domain (residues 417 to 675) was cloned into pCDNA3 with an N-terminal TEV cleavable 6xHIS tag. Protein was expressed in BL21(DE3) cells overnight at 20˚C following induction with 40μM IPTG. The resultant protein was extracted from the cells by sonication in lysis buffer (50mM HEPES pH 8.2, 300mM NaCl, 5% glycerol, 5mM imidazole and 0.5mM TCEP) with a cOmplete EDTA-free protease inhibitor cocktail tablet. Immobilised Metal Affinity Chromatography purification was performed using a 5ml HisTrap™ excel column equilibrated with lysis buffer and elution buffer (50mM HEPES pH 8.2, 300mM NaCl, 5% glycerol, 300mM Imidazole and 0.5mM TCEP). Purified

fractions of BMX kinase domain were further purified using a HiLoad® 16/600 Superdex® 75 pg column equilibrated with gel filtration buffer (20mM HEPES pH 8.2, 150mM NaCl and 0.5mM TCEP). The resulting purified fractions were pooled and treated with TEV protease (1:20 w/w) at 4°C for His-tag cleavage. The samples were purified through Bio-Spin® Chromatography Columns with Amintra Ni-NTA Affinity Resin equilibrated with gel filtration buffer. SDS-PAGE was used to validate protein purity and TEV cleavage. Cleaved protein was concentrated using a centrifugal concentrator with a 10kDa cutoff at 2808 x g to 0.1mg/mL for use in spectroscopy, and to 10mg/mL once secondary structure folding was confirmed. Concentrated protein was stored at -80°C.
