# Supplementary material for: Inhibition of tyrosine kinase BMX increases cell death in response to existing chemotherapeutic agents overcoming apoptotic resistance in cancer
Source: Cell Death Dis. 2025 Nov 10;16(1):813. doi: 10.1038/s41419-025-08131-9 (PMC12603249; doi:10.1038/s41419-025-08131-9)
Supplement: Supplementary file 2 — Raw western blot files [file 41419_2025_8131_MOESM2_ESM.docx]

Figure 3C


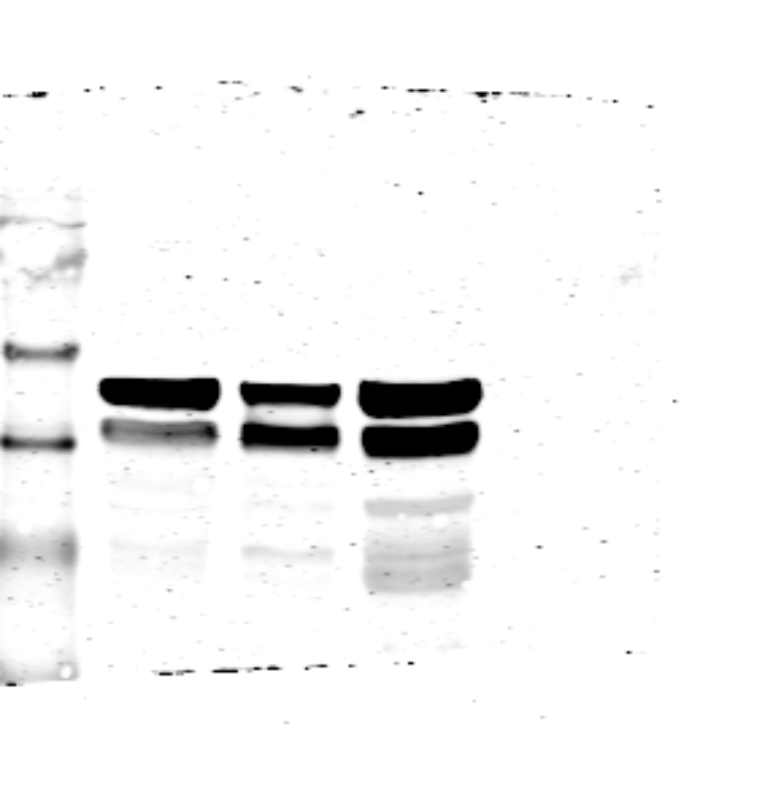


BTK

BMX


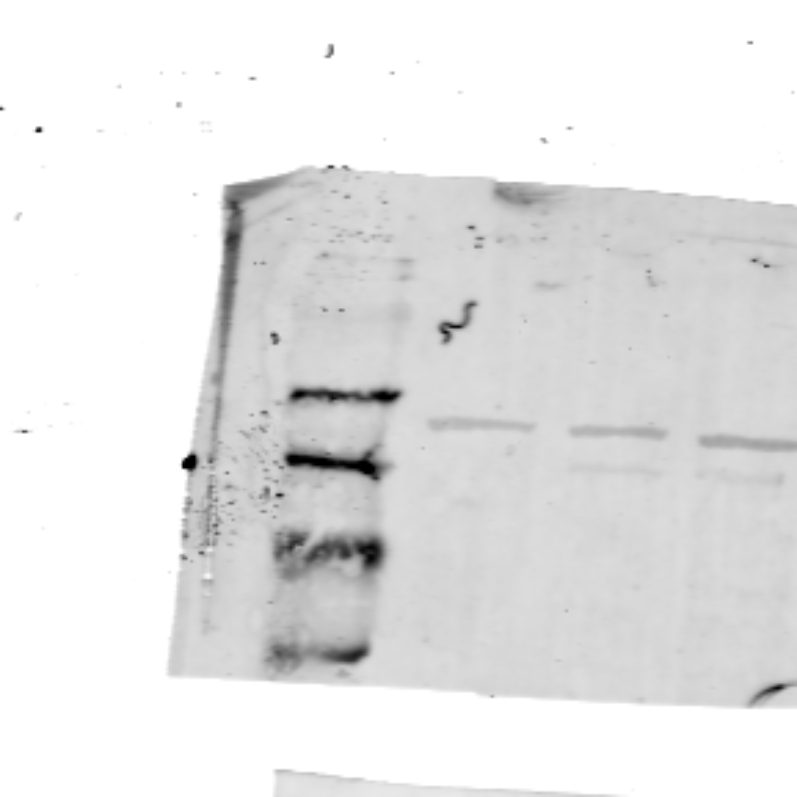


TEC

ITK


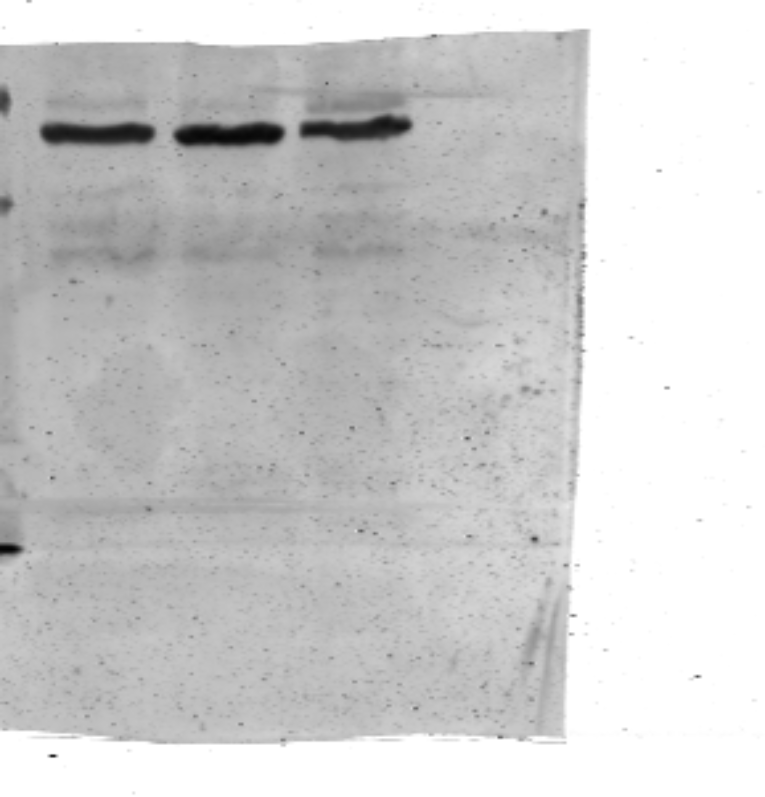


B-actin

Figure 3F


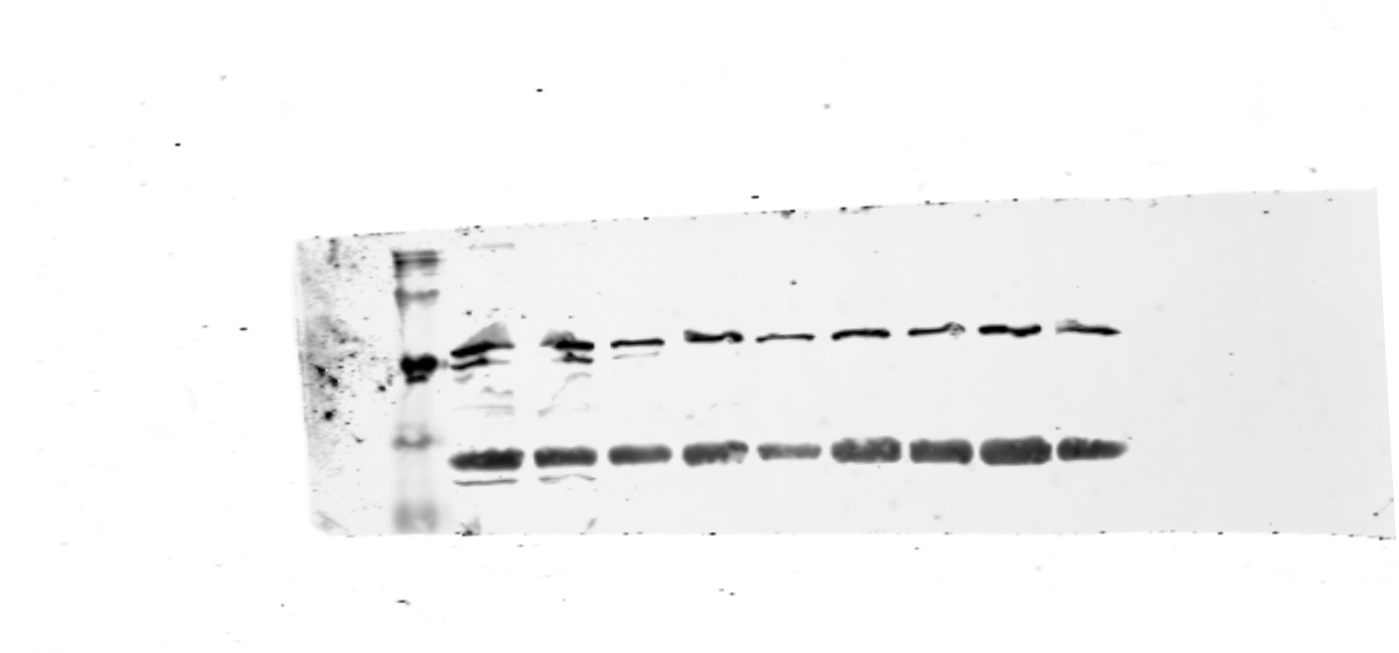


**pAKT =60kDa**


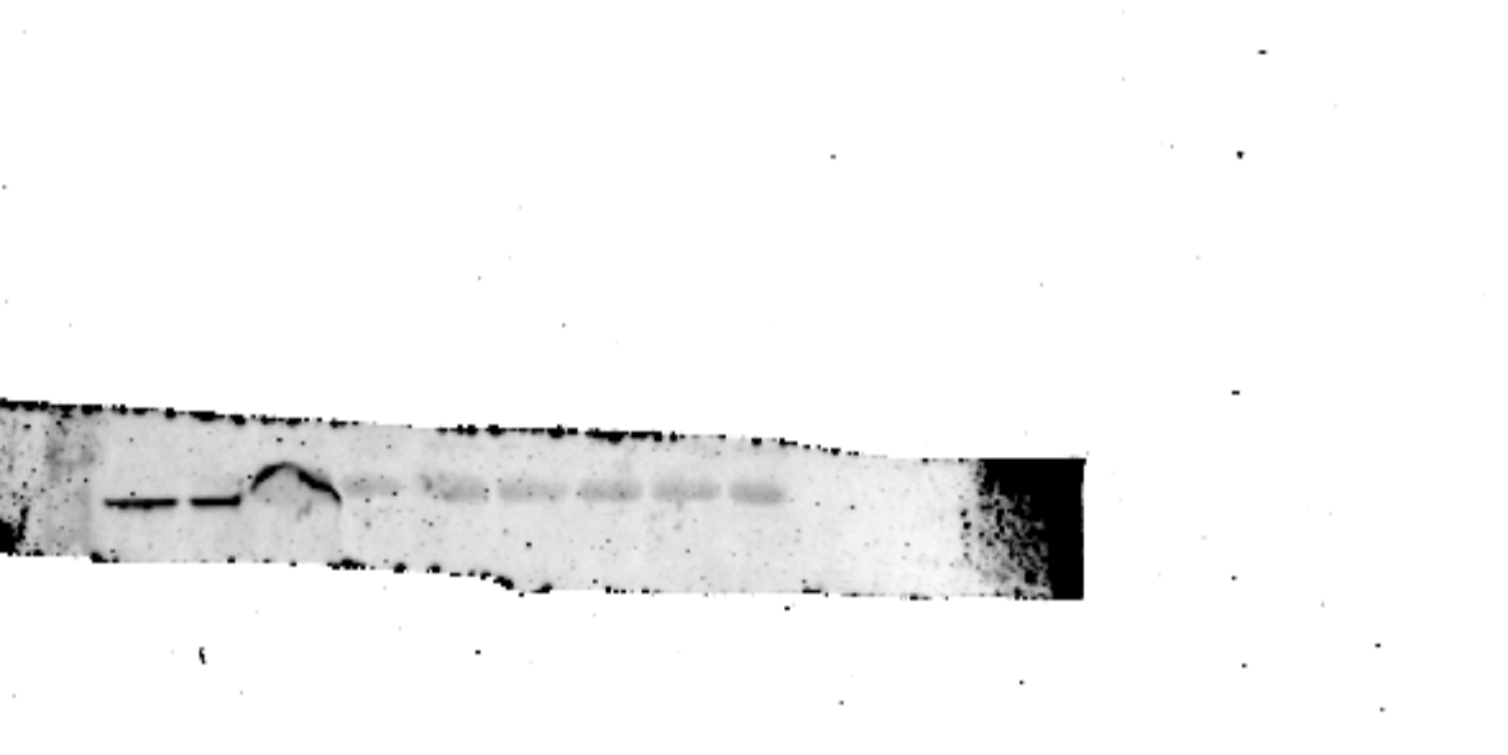


**AKT =60kDa**


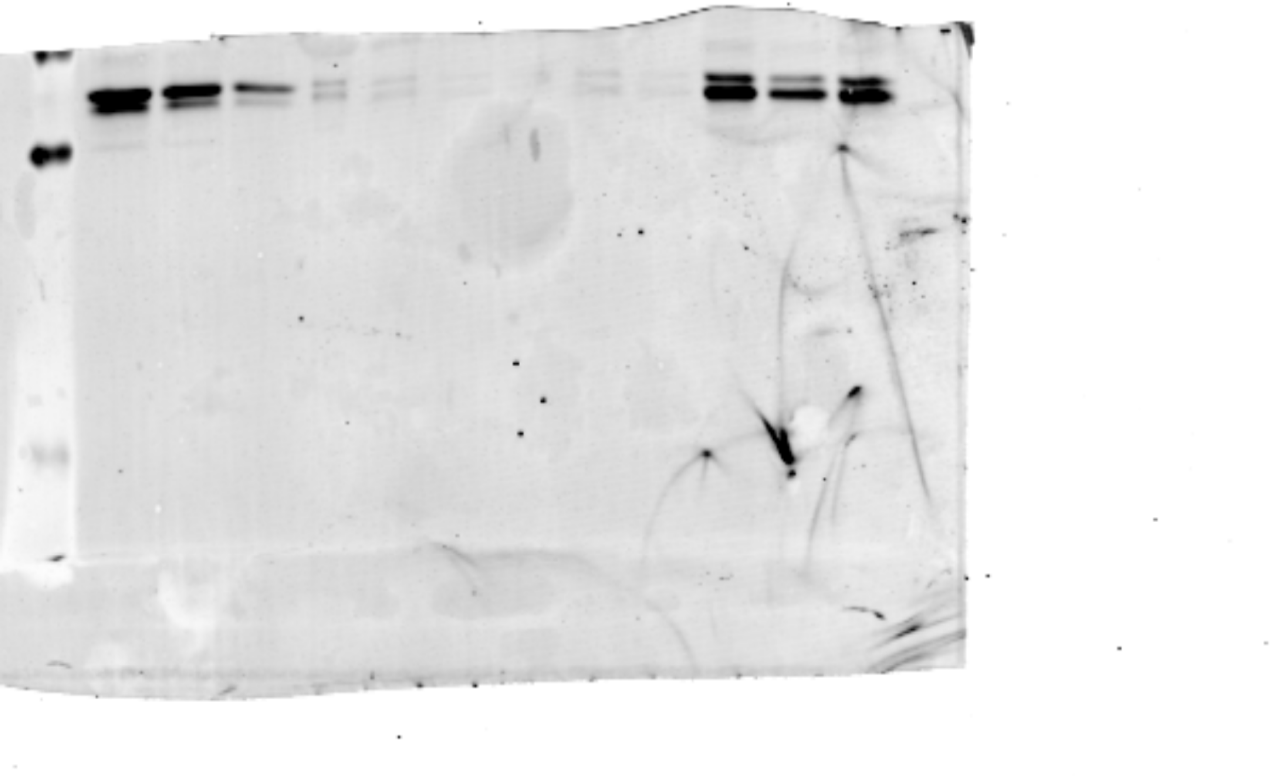


**pERK1/2 =44/42kDa**


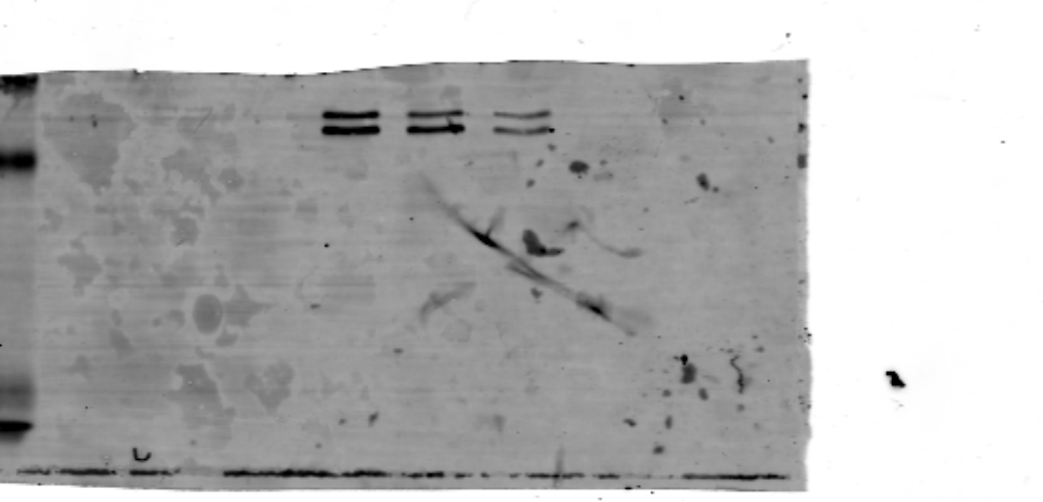


**pERK1/2 =44/42kDa**


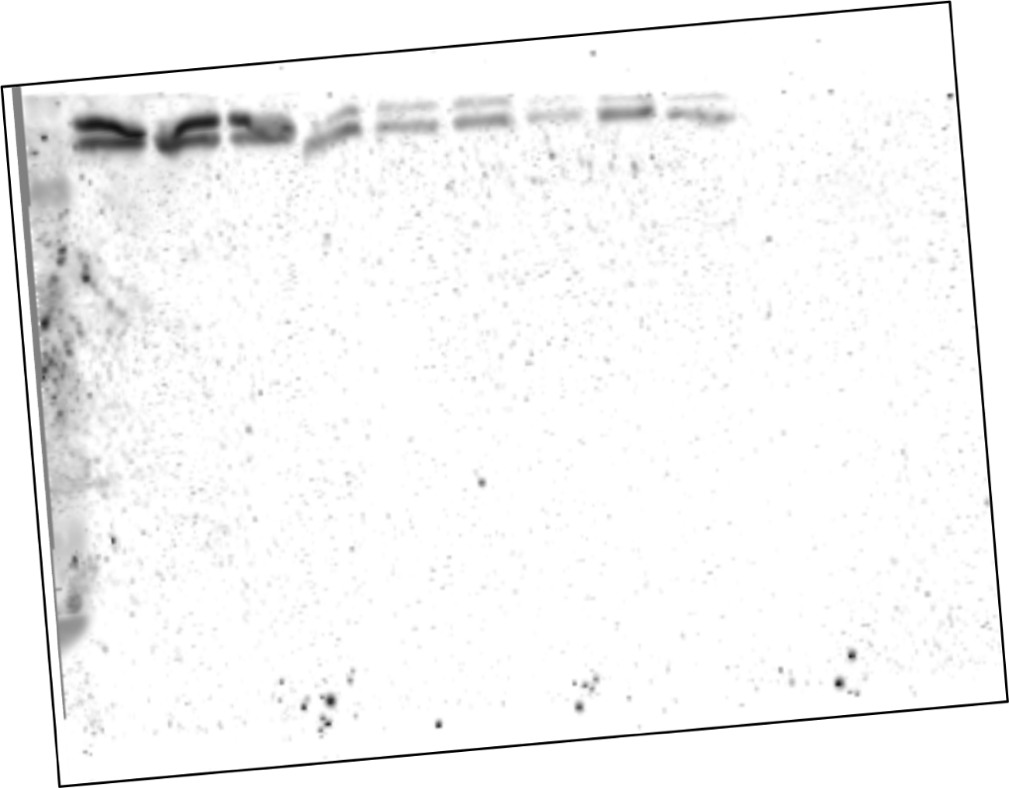


**ERK1/2 =44/42kDa**

**ERK1/2 =44/42kDa**


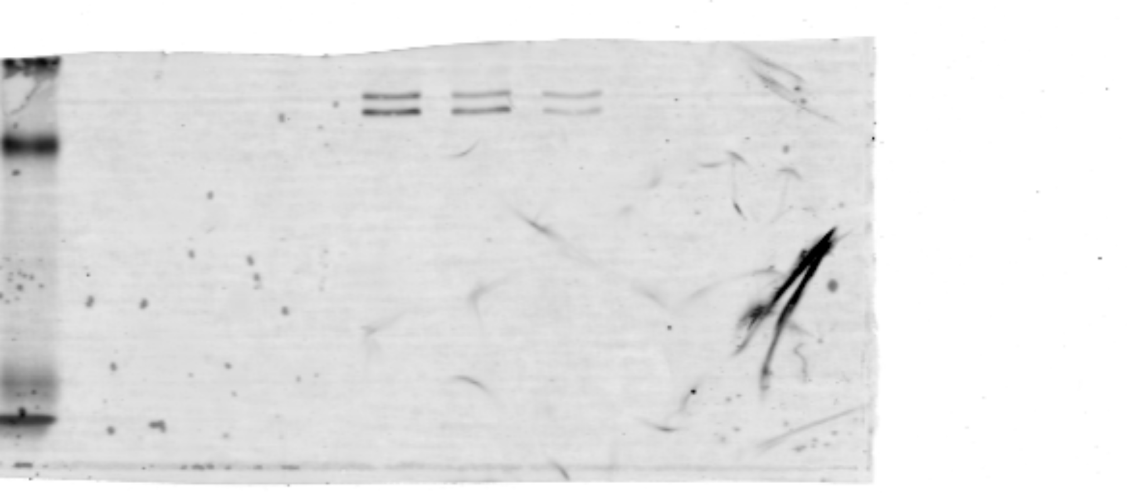


**Vinculin =124kDa**


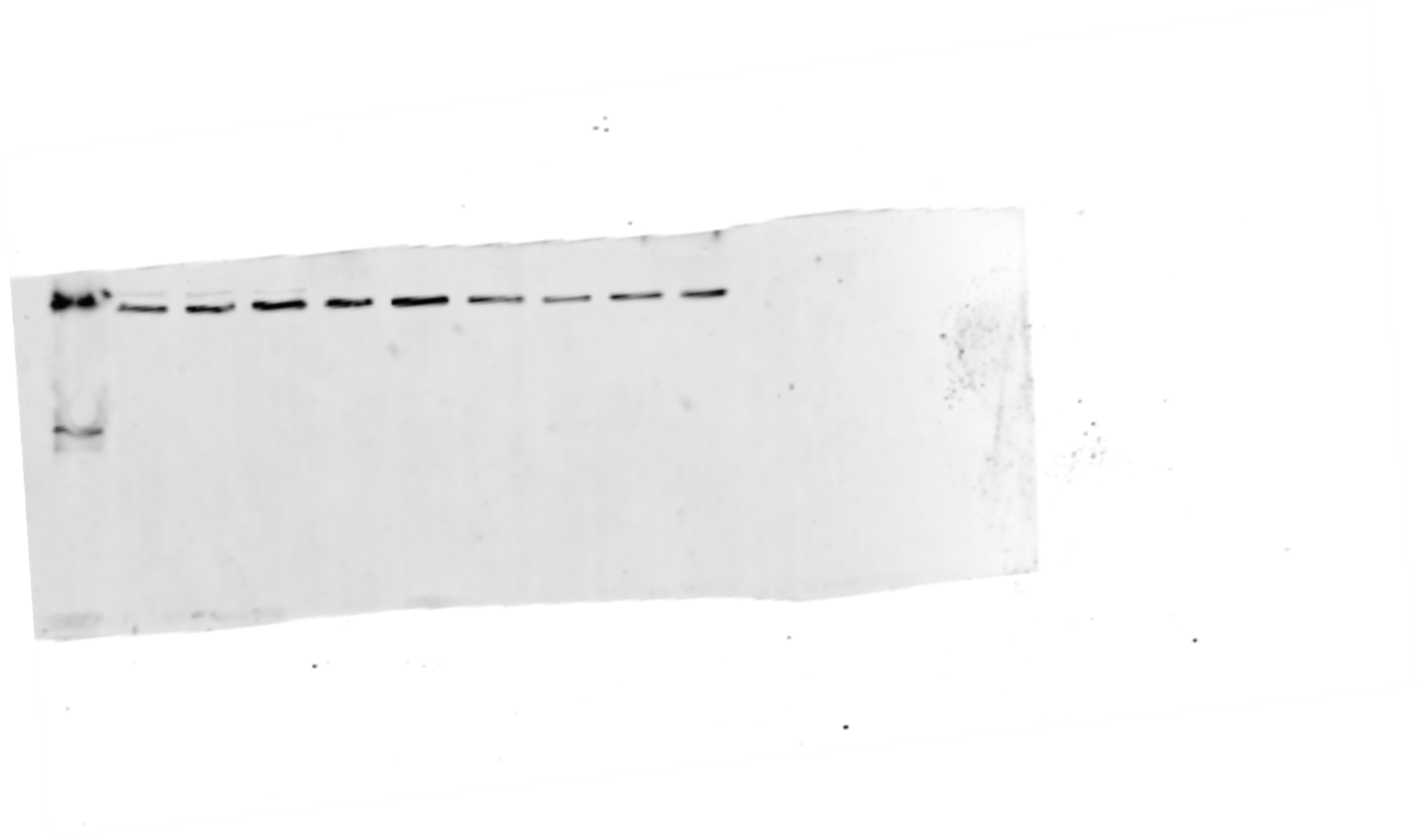


Figure 6E


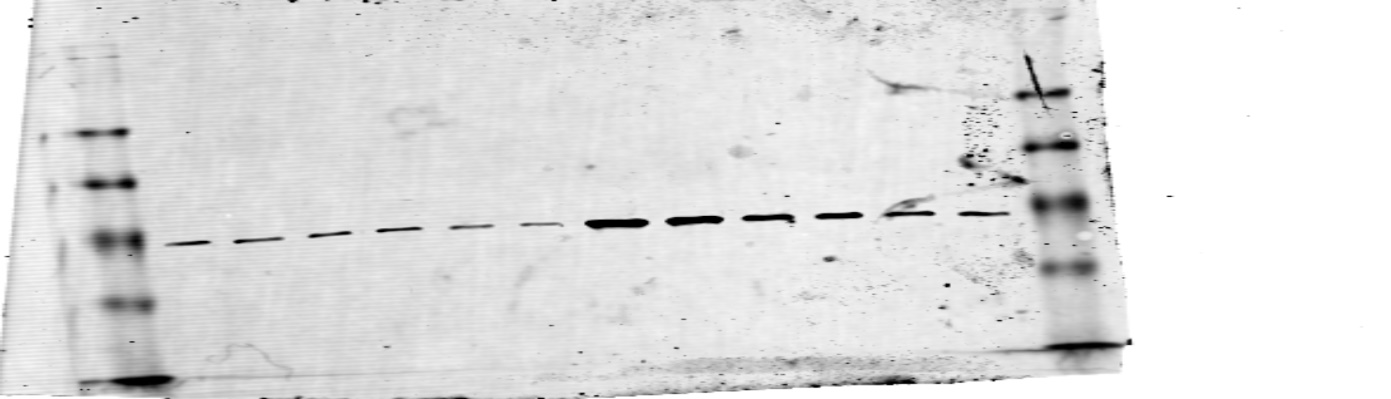


**Vinculin =124kDa**


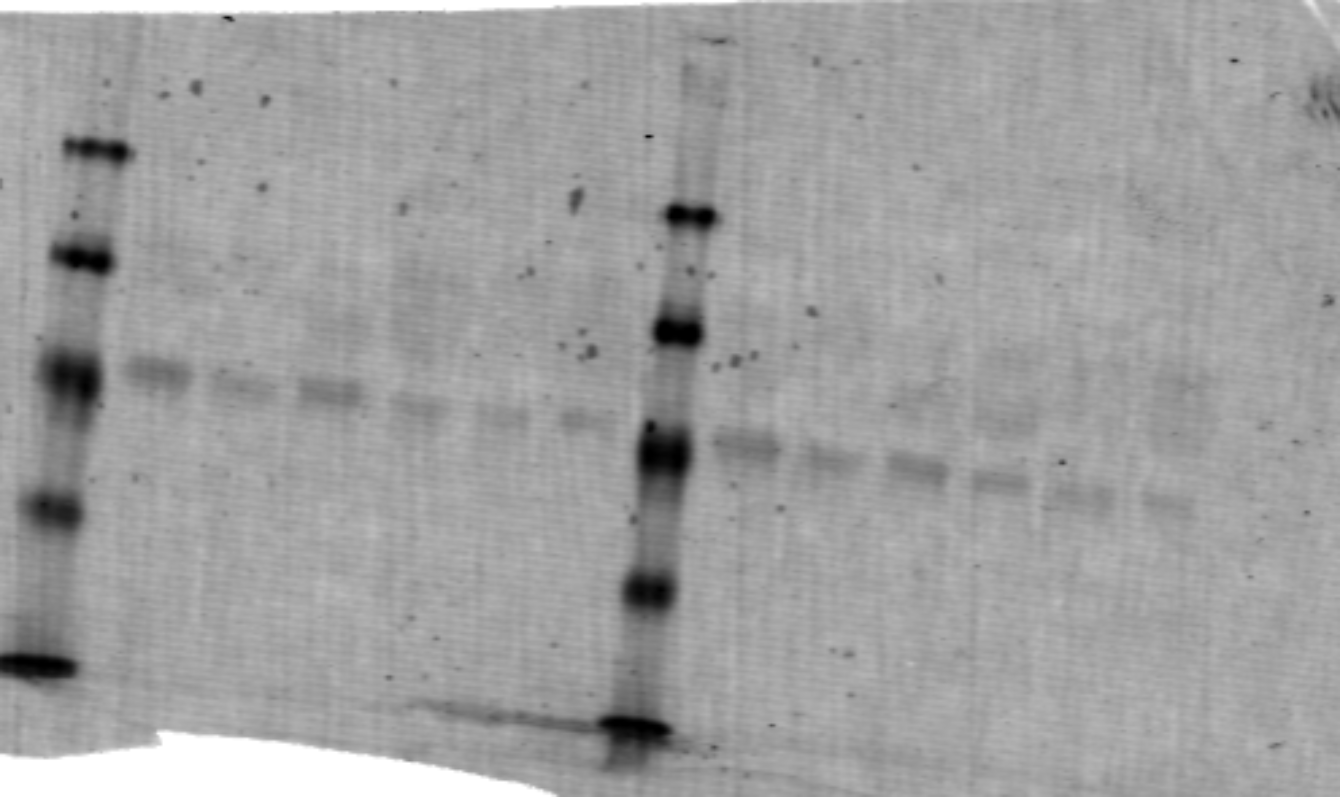


**AKT =60kDa**


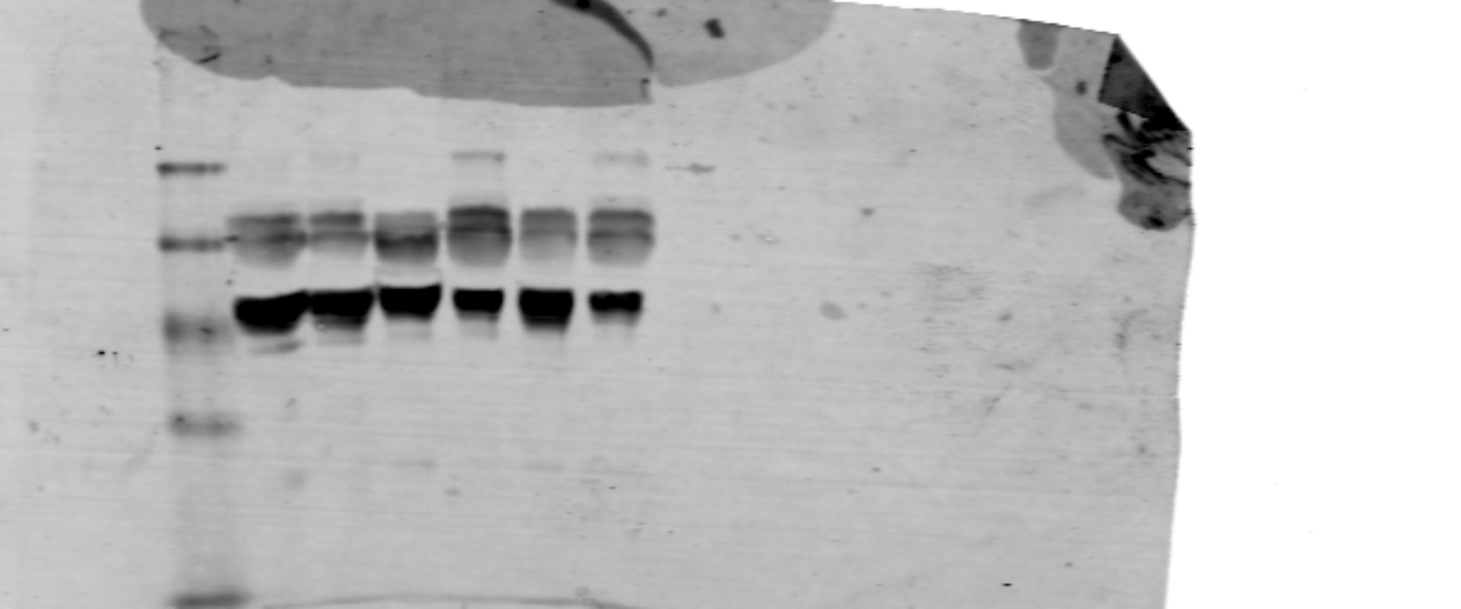


**PAKT =60kDa**


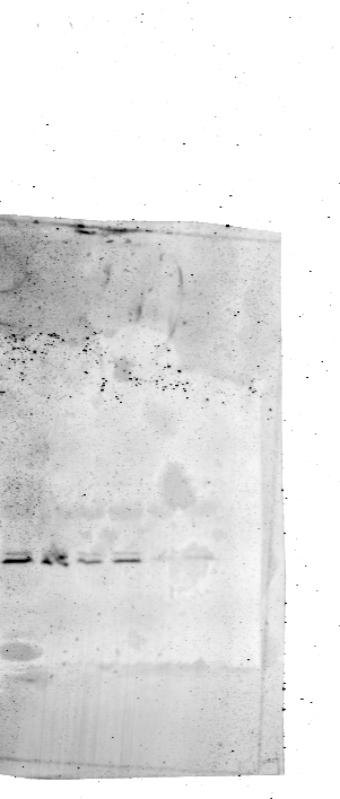


**ERK1/2 =44/42kDa**


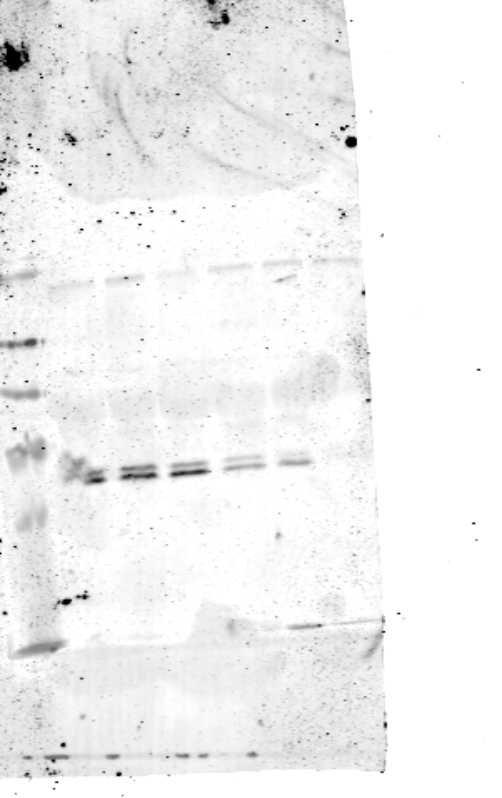


**PERK1/2 =44/42kDa**
